# Analyzing immune response to engineered hydrogels by hierarchical clustering of inflammatory cell subsets

**DOI:** 10.1126/sciadv.abd8056

**Published:** 2022-02-25

**Authors:** Marc A. Fernandez-Yague, Lauren A. Hymel, Claire E. Olingy, Claire McClain, Molly E. Ogle, José R. García, Dustin Minshew, Sofiya Vyshnya, Hong Seo Lim, Peng Qiu, Andrés J. García, Edward A. Botchwey

**Affiliations:** 1 The Wallace H. Coulter Department of Biomedical Engineering, Georgia Institute of Technology and Emory University, Atlanta, GA, USA.; 2 Petit Institute for Bioengineering and Biosciences, Georgia Institute of Technology, Atlanta, GA, USA.; 3 Woodruff School of Mechanical Engineering, Georgia Institute of Technology, Atlanta, GA, USA.

## Abstract

Understanding the immune response to hydrogel implantation is critical for the design of immunomodulatory biomaterials. To study the progression of inflammation around poly(ethylene glycol) hydrogels presenting Arg-Gly-Asp (RGD) peptides and vascular endothelial growth factor, we used temporal analysis of high-dimensional flow cytometry data paired with intravital imaging, immunohistochemistry, and multiplexed proteomic profiling. RGD-presenting hydrogels created a reparative microenvironment promoting CD206^+^ cellular infiltration and revascularization in wounded dorsal skin tissue. Unbiased clustering algorithms (SPADE) revealed significant phenotypic transition shifts as a function of the cell-adhesion hydrogel properties. SPADE identified an intermediate macrophage subset functionally regulating in vivo cytokine secretion that was preferentially recruited for RGD-presenting hydrogels, whereas dendritic cell subsets were preferentially recruited to RDG-presenting hydrogels. Last, RGD-presenting hydrogels controlled macrophage functional cytokine secretion to direct polarization and vascularization. Our studies show that unbiased clustering of single-cell data provides unbiased insights into the underlying immune response to engineered materials.

## Teaser

Predicted by novel bioinformatic tools, cell adhesive forces regulate polyfunctionality of phenotypically similar macrophages.

## INTRODUCTION

The design of immunomodulatory biomaterials represents a fundamental tenet of biomedical device technologies in tissue engineering, wound healing, cancer, and vaccines. In particular, the development of hydrogels that elicit specific host immune responses, facilitating the recruitment of immune components that participate in tissue repair processes, holds considerable promise in immunotherapies and regenerative medicine.

Engineered hydrogels derived from synthetic polymers such as poly(ethylene glycol) (PEG) are particularly attractive because of their cytocompatibility, injectability, and versatility in presenting biomimetic amino acid sequences that encode specific biological responses (*1*–*4*). For example, presenting Arg-Gly-Asp (RGD) peptide, identified first on fibronectin as the minimum cellular integrin-binding motif, facilitates myeloid cell adhesion and supports various immunomodulatory effects such as the decrease in macrophage proinflammatory cytokine production and phagocytosis, generating promising preclinical results (*5*–*7*). However, despite recent progress (*8*), how engineered hydrogels direct immune cell function and heterogeneity after implantation remains largely unexplored, and their clinical translation is yet to be realized.

Under appropriate conditions, the wound healing immune response comprises an inflammatory phase that transitions to a resolution phase (*9*). Innate immune cells are essential in this transition and have proven to be responsive to cues from their microenvironment to orchestrate this reparative transition (*9*–*11*). The functional outcome of biomedical devices is directly correlated with specific phenotypes and functions of the mononuclear phagocyte system (MPS) (*12*, *13*). Components of the MPS, including monocytes, macrophages, and dendritic cells (DCs), play crucial roles in biomaterial-induced tissue repair remodeling with minimal scarring or loss of function (*14*). However, complex, heterotypic interactions among immune cells and injury microenvironment can result in chronic inflammation, nonhealing wounds, fibrosis (*15*), carcinogenesis (*16*), and implant failure (*17*, *18*).

Following injury, monocytes can exist in two principal subsets: proinflammatory early responders defined as classical monocytes and nonclassical monocytes that patrol the resting endothelium and participate in reparative processes after injury (*19*–*21*). As wound healing progresses, monocytes can differentiate into macrophages and display a broad spectrum of gene and protein expression patterns that have been primarily classified as classically activated or “M1” macrophages and alternatively activated “M2” macrophages. However, this is an oversimplification, and new nomenclature has been proposed (*22*). The extracellular matrix microenvironment properties and interactions with other immune cells, including apoptotic neutrophils (*23*) and paracrine signals derived from T helper cells (*14*), may regulate monocyte’s and macrophage’s plastic phenotype and function. Our group has recently demonstrated that adhesive biomaterials can enhance the accumulation of nonclassical monocytes, and their subsequent differentiation into alternatively activated macrophages are associated with enhanced vascularization (*24*) and tissue repair after traumatic injury (*25*). However, CD11b^+^ myeloid cells (monocytes and macrophages) can exhibit significant heterogeneity in cell surface marker expression levels, and new analysis methods to better characterize cell heterogeneity are necessary to begin to understand the immunomodulatory role of engineered biomaterials during wound healing (*26*). Moreover, mass cytometry data sets have markedly increased in size and dimensionality, making it more challenging to decipher meaningful trends (*27*).

Current cell classification methods primarily use biplot gating strategies prone to user error and bias, and only use a fraction of the data available to explore heterogeneity within cell subpopulations (*28*–*33*). The emergence of high-dimensional data reduction algorithms provides solutions to infer additional cellular heterogeneity and function from large data sets. Algorithms such as X-shift and Spanning-tree Progression Analysis of Density-normalized Events (SPADE) are helpful tools to identify emerging cellular heterogeneity while preserving rare cell types without over- or under-clustering, allowing valuable insights into complicated cellular processes to be readily inferred (*32*, *34*). We leverage SPADE in this series of studies to visualize high-dimensional data as a two-dimensional (2D) projection in which each SPADE node represents a cluster of cells similar in phenotype based on a set of protein markers. SPADE then constructs a minimum spanning tree to connect the cell clusters (nodes) into an ordered dendrogram, in which the relative order of nodes may infer cellular transition states at a snapshot in time. These unbiased clustering algorithms can reveal cell heterogeneity and infer cellular transition states, improving the resolution to analyze cellular transitional states both spatially and temporally from the onset of inflammation to resolution.

In the present study, we explored the progression of inflammation around degradable PEG hydrogels presenting adhesive peptides (RGD) and angiogenic growth factor [vascular endothelial growth factor (VEGF)] using SPADE clustering algorithms to analyze high-dimensional flow cytometry data paired with intravital imaging and cytokine secretory profiling. Over 14 days, monocyte and macrophage populations dynamically transitioned from proinflammatory to prohealing phenotypes and promoted dorsal wounded skin tissue revascularization. We demonstrated that vascularization was enhanced by the recruitment of SPADE-identified macrophage subsets, which showed functional heterogeneity in response to the adhesive cues. This study indicates that evaluating immune cell functional homogeneity greatly improves on current observations obtained by traditional immunophenotyping techniques. The results shed light on the importance of macrophage functions in wound healing progression influenced by adhesive engineered hydrogels and demonstrate the advantages of unbiased high-dimensional analysis methods over traditional methods that identify previously excluded populations sensitive to biomaterial adhesive cues.

## RESULTS

### In vivo migration of CX3CR1^+^ mononuclear phagocytes increases in the vicinity of RGD-presenting PEG hydrogels

We engineered degradable PEG hydrogels by cross-linking 2-kDa four-arm PEG macromer containing terminal maleimide groups (PEG-4MAL) with the cysteine-flanked peptide GCRDVPMSMRGGDRCG (VPM) that can be cleaved by proteases such as matrix metalloproteinase–1 (MMP-1) and MMP-2/9 (*35*). To explore how adhesive peptide presentation affected the recruitment of mononuclear phagocytes to implanted hydrogels, PEG-4MAL macromer was functionalized with cysteine-terminated RGD (GRGDSPC) peptide or scrambled peptide control (GRDGSPC, RDG) before hydrogel cross-linking. RGD is a widely used prototypical adhesive peptide derived from fibronectin, which binds to many integrin receptors on cell surfaces such as α_V_β_3_, α_5_β_1_, and α_V_β_1_. RDG serves as its respective nonfunctional control by alternating the residues affecting the binding domain while maintaining the same molecular weight (*36*–*38*). Mixing of adhesive peptide–presenting PEG-4MAL with VPM rapidly forms a hydrogel. Moreover, RGD- and RDG-presenting hydrogels have equivalent network structures, cross-linked densities, and mechanical properties (*39*).

**Fig. 1.**
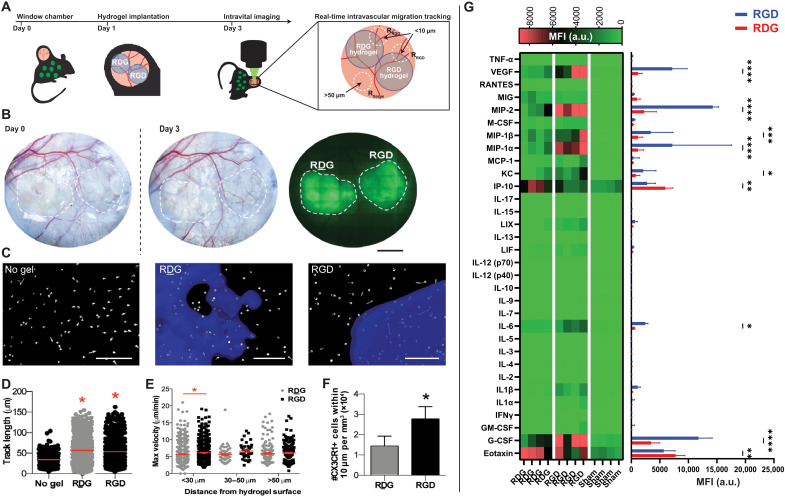
CX3CR1^+^ myeloid cells exhibit modified migration patterns around RGD-functionalized hydrogels. (**A**) The dorsal skinfold window chamber (DSWC) injury was performed on CX3CR1^GFP/+^ mice. Two precast PEG-4MAL hydrogels functionalized with RGD or control RDG peptides were implanted side by side onto the exposed cutaneous tissue (internally controlled design) at 1 day after injury, and intravital imaging was performed on day 3. (**B**) Bright-field images of dorsal tissue at day 0 and day 3 after injury and fluorescent image of tagged hydrogels. Dotted white lines represent location of hydrogel implants (fluorescently tagged hydrogels shown in green). Scale bar, 2 mm. (**C**) Representative renderings of CX3CR1^+^ cell displacement vectors generated during image processing to track cell migration. Hydrogels were fluorescently tagged for detection of the hydrogel edge (blue). Scale bar, 100 μm. Quantifications are made from CX3CR1^+^ cells moving in tissue without a hydrogel or at the interface of hydrogels functionalized with RDG or RGD. (**D**) Track length (μm), (**E**) maximum velocity (μm/min) classified according to cells’ minimum distance from the hydrogel surface, and (**F**) number of CX3CR1^+^ cells within 10 μm of RDG- and RGD-functionalized hydrogels, normalized to hydrogel volume within each image. The red bar indicates the mean (D and E). *P < 0.05 compared to no gel (D) and compared to RDG-presenting hydrogel (E and F) by Kruskal-Wallis with Dunn’s multiple comparisons (D and E) or Mann-Whitney test (F). *n* = 219 to 876 cells across three mice. (**G**) Heatmap visualization of cytokine expression profiles and quantification of lysate extracted from tissues exposed to adhesive (RGD) or nonadhesive (RDG) hydrogels. *n =* 4 animals *P < 0.05 compared to no gel (D) and compared to RDG-presenting hydrogel (E and F) by Kruskal-Wallis with Dunn’s multiple comparisons. **P < 0.01; ***P < 0.005; ****P < 0.001. a.u., arbitrary units.

Immune cell migration is a critical process that mediates the immune response to biomaterials and controls tissue regeneration. To evaluate the effect of the adhesive ligand on the immune cell migratory function, we performed real-time detailed migration analyses of cells on multiple areas around engineered hydrogels implanted in a dermal wound. We used the dorsal skinfold window chamber (DSWC) model, which involves excising the epidermis and outer layers of the dermis, inducing a robust systemic inflammatory response in mice that express green fluorescent protein (GFP) under one copy of the CX3CR1 promoter (CX3CR1^GFP/+^ mice) (*24*). CX3CR1 is expressed primarily not only on monocytes but also on macrophages and DCs (*40*). Following DSWC surgery, we placed pre-cast RDG- or RGD-presenting hydrogels on the exposed subcutaneous layer of the dermis, and 3 days post-implantation (dpi), we performed intravital longitudinal bright-field imaging and laser scanning confocal microscopy at the edge of each hydrogel or in distal tissue lacking a hydrogel (Fig. 1A). In addition, a fluorescent tag was incorporated into the hydrogels during fabrication to visualize the material surface and identify the hydrogel edge (Fig. 1B). Longitudinal bright-field intravital imaging revealed a marked increase in vascular remodeling and expansion at 3 dpi of RGD-presenting hydrogels compared to RDG-presenting hydrogels at day 0 (Fig. 1B).

Using 3D image processing software (Fig. 1C), we found an increase in the average track length around both RDG-presenting (55.7 ± 32.9 μm) and RGD-presenting (53.9 ± 29.1 μm) hydrogels compared to control distal tissue (sham, 33.9 ± 16.4 μm) (Fig. 1D). Then, we performed a classification of cells on the basis of their shortest distance from the hydrogel surface. Most cell migration tracks were within <30 μm of the hydrogel surface, and at that distance, the mean and maximum velocity of CX3CR1^+^ cells toward RGD-presenting hydrogels significantly increased compared to RDG-presenting gels (Fig. 1E). At greater distances (30 to 50 μm and >50 μm), CX3CR1^+^ cell migratory patterns were unchanged by the presence of adhesive ligand on the hydrogel. The velocity of cells moving around RDG-presenting hydrogels was also higher than cells in distal tissue (containing no hydrogel). However, CX3CR1^+^ cells displayed higher mean velocities than in distal tissue for both hydrogel formulations (2.8 ± 0.8 μm/min for distal tissue, 3.6 ± 1.1 μm/min for RDG-presenting hydrogels >50 μm, and 3.9 ± 0.9 μm/min for RGD-presenting hydrogels >50 μm). This distinct trafficking pattern was most significant near the interface of the hydrogels (within <10 μm), where most of the cells preferentially accumulated on RGD-functionalized hydrogel but not on RDG-presenting surfaces (Fig. 1F). These measurements were normalized to the total volume (instead of surface area) of the hydrogel because of the high variability in the surface areas imaged. Overall, these results indicate that CX3CR1^+^ cells migrate differently through interstitial space close to a hydrogel implant than in tissue lacking material. Furthermore, the presentation of RGD ligand in hydrogels affects the tissue response and significantly enhances the migratory activity of cells within 30 μm of the hydrogel surface; however, the same adhesive cues do not affect CX3CR1^+^ cell migration patterns or activity at longer length scales (>30 μm).

We next examined the influence of RGD ligand or its scrambled control RDG presentation in inflammatory cytokine levels at the tissue-hydrogel interface at 7 dpi using the DSWC model. Cells isolated from explanted hydrogels and surrounding tissues were assayed for 32 mouse cytokines using Luminex multiplex technology. As shown in Fig. 1G, the cytokine expression pattern significantly differed between RGD- and RDG-presenting hydrogels [two-way analysis of variance (ANOVA) analysis, *P* < 0.001]. Implantation of RGD- and RDG-presenting hydrogels resulted in increased cytokine levels compared to sham (no hydrogel). Notably, VEGF levels were significantly increased in RGD-presenting hydrogels compared to RDG-presenting hydrogels. Similarly, a significant increase in the production of chemotactic molecules such as granulocyte colony-stimulating factor, monocyte chemotactic protein-1 (MCP-1), and macrophage inflammatory protein-1α and β (MIP-1α and MIP-1β) was observed for cells and tissue interfacing with RGD-presenting hydrogels. We postulate that as cells adhere to the hydrogel surface, they secrete chemokines, creating a gradient that stimulates speed of cells, particularly those near the surface. Furthermore, interleukin-6 (IL-6) and CXCL1/keratinocyte-derived chemokine (KC), which play diverse immunomodulatory roles, including recruitment and polarization of macrophages, were up-regulated in RGD-functionalized hydrogels compared to RDG-functionalized hydrogels. In contrast, levels of interferon gamma (IFN-γ)–induced protein 10 (IP-10) and eotaxin, which activate multiple proinflammatory cascades, were up-regulated for cells isolated from RDG-presenting gels compared to RGD-presenting hydrogels (Fig. 1G). Overall, these results are consistent with the observed increase in vascular remodeling, cell migration, and cytokine concentrations induced by RGD-functionalized hydrogels and suggest that adhesive cues modulate important immune cell functions, resulting in changes in immune cell migration and cytokine responses.

### UMAP analysis shows temporal myeloid cell recruitment and phenotypic changes to PEG hydrogels

**Fig. 2.**
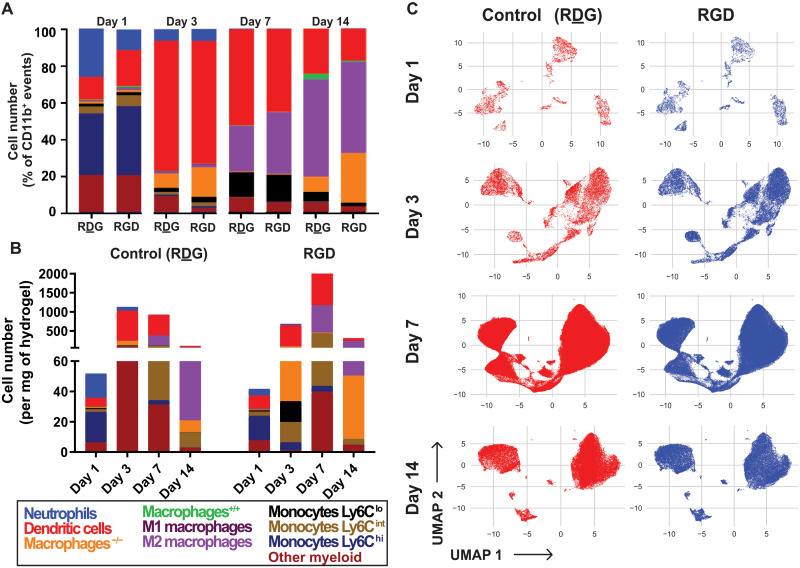
UMAP dimensionality reduction showed temporal shifts in immune cell recruitment and predominant macrophage recruitment during the resolution phase. (**A**) Frequency of myeloid immune cell populations from CD11b^+^ events at days 1, 3, 7, and 14 after injection into the subcutaneous space. Myeloid immune cell subsets include neutrophils, DCs, macrophages^++^ (CD86^+^ CD206^+^), M1 macrophages (CD86^+^ CD206^−^), M2 macrophages (CD86^−^ CD206^+^), macrophages^− −^ (CD86^−^ CD206^−^), Ly6C^lo^ monocytes, Ly6C^int^ monocytes, Ly6C^hi^ monocytes, and ungated myeloid cells. (**B**) Distribution of myeloid immune cell populations normalized to explanted tissue/hydrogel weight at days 1, 3, 7, and 14 after injection. (**C**) UMAP projections of cellular infiltrate at days 1, 3, 7, and 14, respectively, for RDG-functionalized hydrogels (left) and RGD-presenting hydrogels (right). *n =* 3 to 4 animals per time point with internally controlled design.

Because of the recognized importance of myeloid cells (monocytes and macrophages) in the functional outcomes of implanted biomaterials, we investigated the kinetics of myeloid cell recruitment and phenotypic changes in response to adhesive hydrogels in a subcutaneous implantation site using multiparametric flow cytometry. Hydrogel precursors were injected and polymerized in situ within the dorsal subcutaneous space of mice. Each mouse received two hydrogel formulations: a control hydrogel functionalized with the scrambled peptide RDG on the left side and RGD-functionalized hydrogel on the right side. This design controls mouse-mouse variance and ensures a more accurate comparison of single-cell events recruited to the hydrogels as a function of adhesive ligand per mouse replicate. At 1, 3, 7, or 14 dpi, hydrogels were explanted and digested with collagenase to extract hydrogel-associated cells for immunophenotyping by multiparametric flow cytometry. Various distinct cell populations responded to hydrogel implantation, including myeloid cells such as macrophages (CD64^+^ MerTK^+^), DCs (CD11c^+^), neutrophils (Ly6G^+^), and monocytes (CD64^+^ CD11b^+^) that were quantified using a traditional gating strategy on FlowJo software (fig. S1). Macrophage subsets (M1, M2, M^−/−^, and M^+/+^) and monocyte subsets (Ly6C^hi^, Ly6C^int^, and Ly6C^lo^) were further gated on the basis of their surface expression of CD86 and CD206 or their differential expression of Ly6C, respectively (fig. S2). To explore the heterogeneity of inflammatory cells recruited to hydrogels, the composition of CD11b^+^ myeloid cells was evaluated for RGD- and RDG-presenting gels for each time point (Fig. 2A and fig. S3, A to I). At 1 dpi, the presentation of RGD to the hydrogel did affect neutrophil frequency compared to RDG-presenting hydrogels (11.7 ± 5.8% versus 3.9 ± 2.1%, *P* = 0.036), whereas for 3, 7, and 14 dpi, neutrophil counts remained low and no differences between RDG-and RGD-presenting hydrogels were observed (fig. S3A). In contrast, significantly more DCs were recruited to RGD-presenting hydrogels at 1 dpi than RDG-presenting hydrogels (19.5% versus 12.3%, *P* = 0.012) (Fig. 2A, fig S3A). On days 7 and 14, RDG-functionalized hydrogels had a higher frequency of CD11b^+^ events that did not fall into traditional biplot gates, referred to here as “other myeloid cells,” compared to RGD-presenting hydrogels (8.3 ± 1.6% versus 5.7 ± 1.0% at day 7, *P* = 0.019; 5.7 ± 1.4% versus 3.4 ± 1.7% at day 14, *P* = 0.011) (Fig. 2A and fig S1b). Last, at 3 and 14 dpi, double-lo macrophages were more accumulated in RGD-presenting hydrogels (16 ± 12.2% versus 7.9 ± 6.3% at day 3, *P* = 0.049 and 27 ± 14% versus 8.3 ± 5.4% at day 14, *P* = 0.046), whereas M^+/+^ macrophages were more frequent in RDG-presenting hydrogels at day 14 (0.5% versus 2.9%, *P* = 0.026). All other comparisons did not reveal differences in CD11b^+^ myeloid cell frequency to the different hydrogels. To determine whether the presentation of RGD peptide affected the counts of immune cells recruited to the hydrogel, the cell number per milligram of tissue was plotted per day for each gel formulation (Fig. 2B and fig. S3, J to R). The total number of CD11b^+^ cells recruited to RGD-presenting gels was significantly higher at 7 dpi (1984 ± 1059 cells/mg versus 993 ± 401 cells/mg, *P* = 0.003) demonstrating the cell adhesion impact of the RGD-presenting hydrogel. Specifically, this effect was associated with an increase of M2 macrophages (652 ± 347 cells/mg versus 265 ± 165 cells/mg, *P* = 0.014) and DCs (963 ± 538 cells/mg versus 504 ± 73 cells/mg, *P* = 0.001 at day 7) significantly recruited toward RGD-presenting hydrogels. Together, the analysis of the myeloid populations identified from the FlowJo gating strategy in traditional bulk quantities revealed minor differences among immune cells recruited to the RGD-presenting hydrogels compared to hydrogels presenting its inactive control, RDG (fig. S3, A to R). To visualize the overall heterogeneous immune response to the distinct hydrogel systems, we performed Uniform Manifold Approximation and Projection (UMAP) for nonlinear dimension reduction of multiparametric flow cytometry data at 1, 3, 7, and 14 dpi (Fig. 2C). Unlike other data reduction techniques [t-distributed stochastic neighbor embedding (tSNE)], UMAP preserves the global structure of local relationships within cell populations (expressing a characteristic surface marker profile). UMAPs were generated from single-cell events extracted from both RGD- and RDG-functionalized hydrogels at each flow cytometry time point (day 1, 3, 7, or 14). Thus, each UMAP illustrates the topological structure of the data at a single time point where single cells are plotted within “islands” of similar marker expression values (Fig. 2C). Comparing between RGD- and RDG-presenting hydrogel formulations for each day after hydrogel implantation, UMAP did not reveal clear differences between the two hydrogel formulations in terms of recruited immune cell clusters, as the cells from RGD-presenting hydrogels (blue) appear to be in the same dimensional space as those from RDG-functionalized hydrogels (red). Overall, we saw a characteristic increase in the frequency of CD206^+^ macrophages and a decrease of DCs over time for RDG- and RGD-presenting hydrogel. Whereas temporal shifts in myeloid populations’ heterogeneity and recruitment frequency were observed for RGD- and RDG-presenting hydrogels, the analyses based on traditional gating strategy and UMAP did not reveal more complex, functionally relevant subpopulations associated with the adhesive ligand presentation.

### Controlled VEGF delivery alters myeloid response to hydrogel implantation

**Fig. 3.**
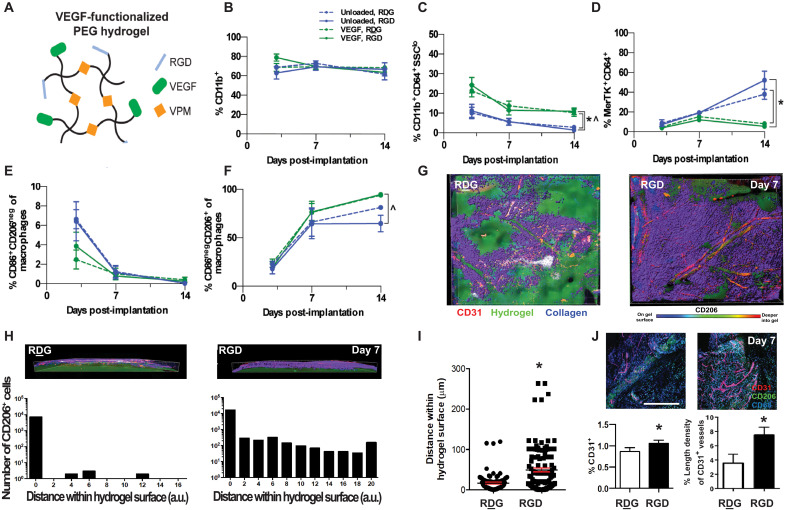
VEGF-loaded RGD-presenting hydrogels promotes vascularization and macrophage cell infiltration. (**A**) Schematic representation of four-arm PEG-MAL macromers functionalized with RGD (or control RDG) peptides and cross-linked with VPM protease–degradable peptide. (**B** to **H**) Immune cell subsets quantified by flow cytometry at 3, 7, and 14 dpi into subcutaneous space. All quantifications are normalized to percentage of single cells between unloaded hydrogels (blue) and VEGF-loaded hydrogels (green), functionalized with either RGD (solid line) or RDG (dotted line) peptides. Immune cells analyzed include CD11b^+^ myeloid cells (B), monocytes (C), macrophages (D), M1 macrophages (E), and M2 macrophages (F). Data expressed as means ± SEM *P < 0.05 compared to unloaded RDG-presenting hydrogel, ^*P* < 0.05 compared to unloaded RGD-presenting hydrogel by one-way ANOVA at the indicated time point. *n =* 3 to 11 hydrogels per group. (G) Immunohistochemistry for CD31 (red) and CD206 (purple) was performed 7 dpi of VEGF-loaded hydrogels in DSWCs. Collagen was visualized using second harmonic generation with multiphoton microscopy (blue). The hydrogel was visualized by fluorescently tagging the ligands (RGD and RDG) with Alexa Fluor 405 (green). (H) Quantitative analysis of the number of cells in function to the distance to the hydrogel surface. A significant increase in the number of CD206^+^ cells is observed at the proximity of the hydrogel surface. (**I**) Quantitative analysis of the accumulated number of cells near the hydrogel surface. (**J**) Quantitative analysis of blood vessel length normalized to the analysis region’s area. Scale bar, 100 μm. Data expressed as means ± SEM *P < 0.05 by paired *t* test. *n =* 102 cells across six animals per group.

Next, we examined myeloid cell accumulation around hydrogels delivering the angiogenic growth factor VEGF, which has been explored for promoting therapeutic vascularization and increasing implant integration with the host (*41*–*43*). VEGF was tethered into the hydrogel network to be slowly released as cells infiltrate and proteolytically degrade and remodel the synthetic matrix. Each mouse received two subcutaneously implanted hydrogels that were either unloaded or loaded with 250 ng of VEGF. We compared data collected from animals receiving VEGF-loaded RGD- and RDG-presenting hydrogels to data collected from animals receiving unloaded RGD- and RDG-presenting hydrogels (Fig. 3A). The frequency of CD11b^+^ myeloid cells was similar across all groups, indicating that neither VEGF nor RGD adhesive ligand affects recruitment of total myeloid cells (Fig. 3B). Ly6G^+^ neutrophils were present at the highest frequency 3 dpi and decreased by 7 dpi in all groups. Whereas the average percentage of neutrophils appeared higher in both VEGF-loaded hydrogels, we detected no significant differences (*P* > 0.05, ANOVA). The frequency of nonclassical CD64^+^ SSC^lo^ monocytes was higher for VEGF-loaded RDG- and RGD-presenting hydrogels than unloaded hydrogels at 14 dpi (Fig. 3C). We also detected fewer MerTK^+^CD64^+^ macrophages around both VEGF hydrogel formulations compared to unloaded hydrogels at day 14 but not at earlier time points (Fig. 3D). While the frequency of CD11c^+^ DCs around unloaded hydrogels decreased from days 7 to 14, no decrease was observed around VEGF-loaded hydrogels, and the frequency of DCs was higher around VEGF-loaded hydrogels at day 14. This result indicates persistence of DCs at day 14 induced by VEGF delivery.

We detected no effect on monocyte composition in response to VEGF delivery or RGD presentation (fig. S4). The proportion of macrophages that were CD86^+^ CD206 (M1-like) was highest for all hydrogel formulations at 3 dpi (between 2 and 7%) but rapidly decreased over 14 dpi (<1%) and was not significantly different among the groups (Fig. 3E), whereas the proportion of CD86^−^ CD206^+^ macrophages increased from 3 to 14 dpi (Fig. 3F). At 14 dpi, we detected a more significant proportion of CD86^−^ CD206^+^ macrophages around RGD-functionalized VEGF hydrogels than unloaded RGD-presenting hydrogels (Fig. 3F). These results indicate that VEGF delivery from PEG hydrogels primarily affects the composition of myeloid cells at later time points (day 14) and that RGD presentation does not affect total cell recruitment regardless of VEGF delivery.

Last, we investigated whether RGD presentation promotes localized vascularization in the presence of VEGF by enhancing the adhesion and infiltration of the “protissue reparative” M2-like macrophages (alternatively activated macrophages, CD206^+^) (Fig. 3G). We coimplanted RGD- and RDG-presenting hydrogels (loaded with VEGF) adjacent to each other using the DSWC in the CX3CR1^GFP/+^ mice and evaluated vessel growth and CD206^+^ cell number by confocal microscopy at 7 dpi. Using 3D image processing, we found that regions of CD206^+^ cell infiltration were associated with hydrogel degradation and vascular ingrowth into the RGD-presenting hydrogel, but this effect was not evident for RDG-presenting hydrogels (Fig. 3G). Furthermore, in contrast to RDG-presenting hydrogels, the number of CD206^+^ cells near RGD-presenting hydrogels was significantly increased (Fig. 3, H and I). We then used whole-mount immunohistochemistry to visualize new microvessels (CD31) (Fig. 3J). To quantify the extent of vascularization, we selected the region of greatest vessel density within each hydrogel and measured the length of CD31^+^ blood vessels. New vessels growing in the tissue surrounding the hydrogels were generally seen, with a higher blood vessel density at the tissue-material interface. In addition, larger and more regular blood vessels grew around VEGF-loaded RGD-presenting hydrogels (Fig. 3J), whereas VEGF-loaded RDG-presenting hydrogels showed fewer and smaller vessels, demonstrating that presentation of cell adhesive cues on PEG hydrogels promotes blood vessel growth at the tissue-hydrogel interface. Together, these results demonstrate that the copresentation of VEGF and RGD at the tissue-hydrogel interfaces promotes recruitment of pro-repair immune cell populations and vascularization. Furthermore, the increase of CD86^−^ CD206^+^ M2-like macrophages at 14 dpi around RGD-functionalized VEGF-loaded hydrogels suggests a role for M2-like macrophages in VEGF-induced vascularization.

### Cell adhesion cues regulate the polyfunctionality of macrophages

**Fig. 4.**
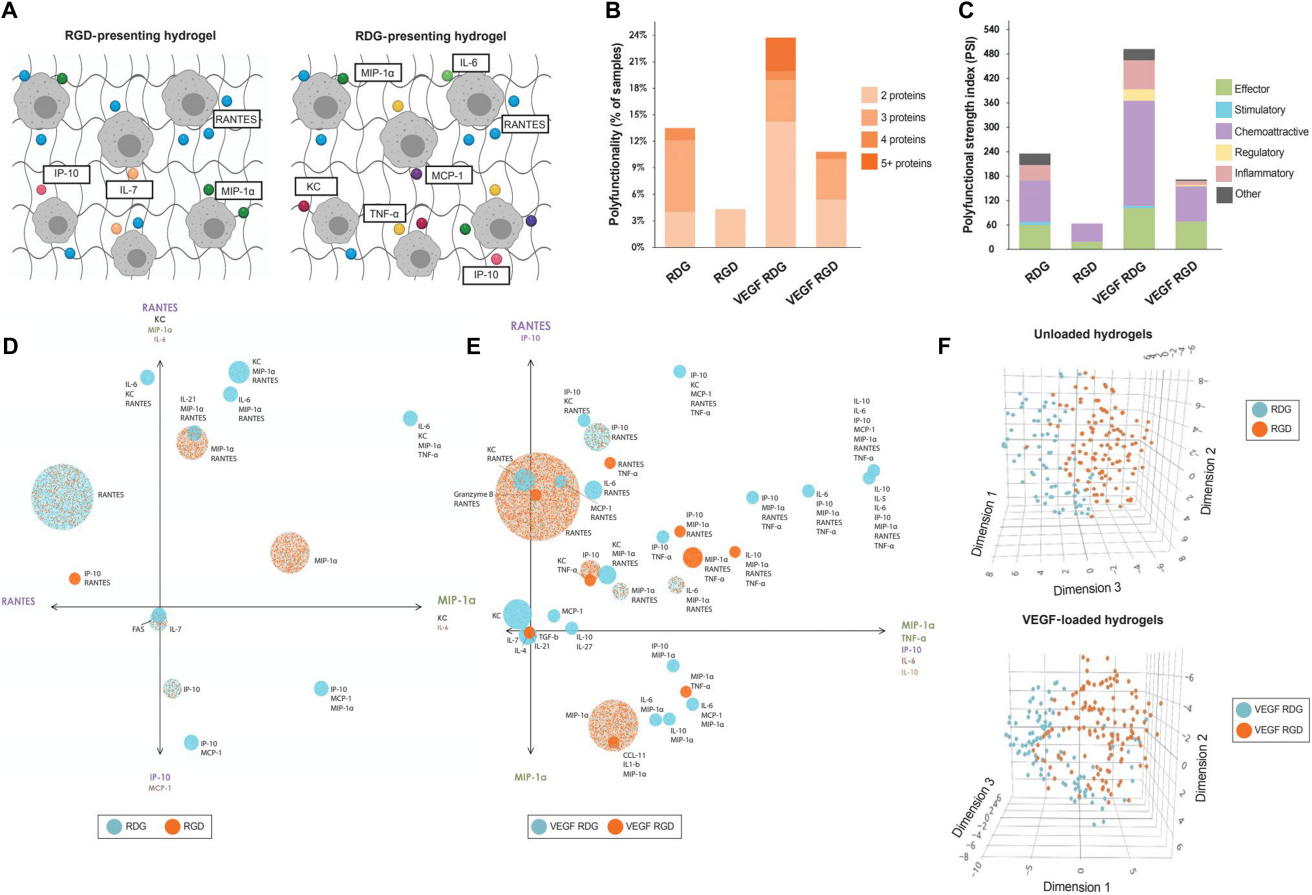
RGD-presenting hydrogels stimulate specific macrophage cytokine signatures. MerTK^+^CD64^+^ macrophages were FACS-sorted from RGD- or RDG-functionalized hydrogels with or without VEGF at 7 dpi. Sorted macrophages were loaded into IsoCode chips for single-cell cytokine analysis. (**A**) Schematic representation of cytokine secreting macrophages from RGD- or RDG-functionalized hydrogels. (**B**) Polyfunctionality or heterogeneity of single macrophages from each hydrogel formulation. Polyfunctional macrophages secrete two or more different cytokines. (**C**) Polyfunctional strength index (PSI) of macrophages from each treatment group. The PSI combines the polyfunctionality of the sample with the average intensity of the analytes secreted by single cells. Each bar is broken down by the PSI of each cytokine class grouping. (**D** and **E**) Polyfunctional activation topology PCA plot representing each polyfunctional group. Each circle corresponds to an individual group, and the analytes labeled next to it represent which analytes are in the functional group. The overall color of the circle indicates which hydrogel formulation secreted this functional group with the highest frequency. Analytes listed at the ends of each axis depict the analytes most strongly present in each principal component. (**F**) 3D t-SNE plots of single macrophages from RGD-functionalized (orange) and RDG-functionalized (blue) hydrogel samples. Default parameters are as follows: theta = 0.5, perplexity = 50, iterations = 1000.

As we noted no major alterations in myeloid phenotype as a response to the adhesive ligand that could correlate with the differing observed tissue responses, we further characterized how the adhesive ligand influences the cytokine microenvironment and function of macrophages. We performed single-cell proteomics analysis of fluorescence-activated cell sorter (FACS)–sorted CD64^+^ MerTK^+^ cells (macrophages) collected from explanted unloaded and VEGF-loaded hydrogels functionalized with either RGD or RDG at 7 dpi. This technique allows for the quantification of cytokine secretions with a broad range of functional profiles and creates a unique polyfunctional metric [polyfunctional strength index (PSI)], which differs from the traditional (flow cytometry–based) intracellular protein measurement. This proteomic evaluation demonstrated that the polyfunctionality of single macrophages is notably elevated in RDG-presenting hydrogels (with or without VEGF) as individual macrophages secreted up to four different cytokines simultaneously, whereas macrophages isolated from explanted VEGF-loaded RDG-presenting hydrogels secreted up to five or more different cytokines (Fig. 4, A and B). In contrast, RGD presentation resulted in macrophages with modulated polyfunctionality in both unloaded and VEGF-loaded hydrogels (Fig. 4, A and B), suggesting that the RGD ligand plays a significant role in determining macrophage polyfunctionality in the immune response to implanted biomaterials (Fig. 4A).

Numerous polyfunctional groups are identified, classified as RGD or RDG (with or without VEGF) polyfunctional groups, each secreting a particular combination of cytokines. Each group is represented by a circle in the polyfunctional activation topology principal components analysis (PCA) plot (Fig. 4D), and each color corresponds to a different hydrogel formulation. The size of the circles corresponds to the frequency of the cytokine group. The two principal components PC1 and PC2 are a combination of the dominant cytokines that drive the polyfunctionality of macrophages. For both graphs, PC2 is a combination of inflammatory (IL-6, MCP-1) and effector cytokines (MIP-1α, TNF-α), whereas PC1 mainly represents chemoattractive cytokines (RANTES, KC, IP-10).

Macrophages isolated from RGD-presenting (RGD) hydrogels secreted functional homogeneous cytokine groups of <2 cytokines with an effector (MIP-1α) and chemoattractive (IP-10, RANTES) functions (Fig. 4, C and D). Furthermore, VEGF-loaded RGD-presenting hydrogels stimulated further the macrophages to secrete up to 4 cytokines with stimulatory (IL-21), chemoattractive (RANTES, CCL11, KC, IP-10), regulatory (IL-4, IL-10, TGF-β), and effector (Granzyme B, TNF-α, MIP-1α) functions ( Fig. 4, C and E). In contrast, macrophages isolated from RDG-presenting hydrogels with or without VEGF showed marked polyfunctional heterogeneity (4 and ≧ 5 respectively, Fig. 4, C, D and E). The macrophage response to RDG-presenting hydrogel added pro-inflammatory (IL-6, MCP-1α, IL-1β) and stimulatory (IL-5, IL-7) functions. These results suggest that RGD presentation directs the phenotype of macrophages shifting from heterogenous pro-inflammatory to more homogenous chemoattractive and effector function. In addition, VEGF incorporation into adhesive hydrogels increased chemoattractive and effector macrophage functions, in correspondence with the observed increased CD206^+^ macrophage infiltration into the RGD hydrogels (Fig. 4E). Together, in-depth cytokine profiling of single macrophages from RGD- and RDG-presenting hydrogels reveals clear differences in the immune response following a function of adhesive ligand presentation (Fig. 4F). Although these underlying differences in the immune response are easily overlooked in flow cytometry cell frequency analysis, these RGD-dependent macrophage secretions may significantly alter autocrine and paracrine signaling within the microenvironment crucial to the wound healing process.

### SPADE trajectory analysis unravels underlying myeloid phenotypic transitions

**Fig. 5.**
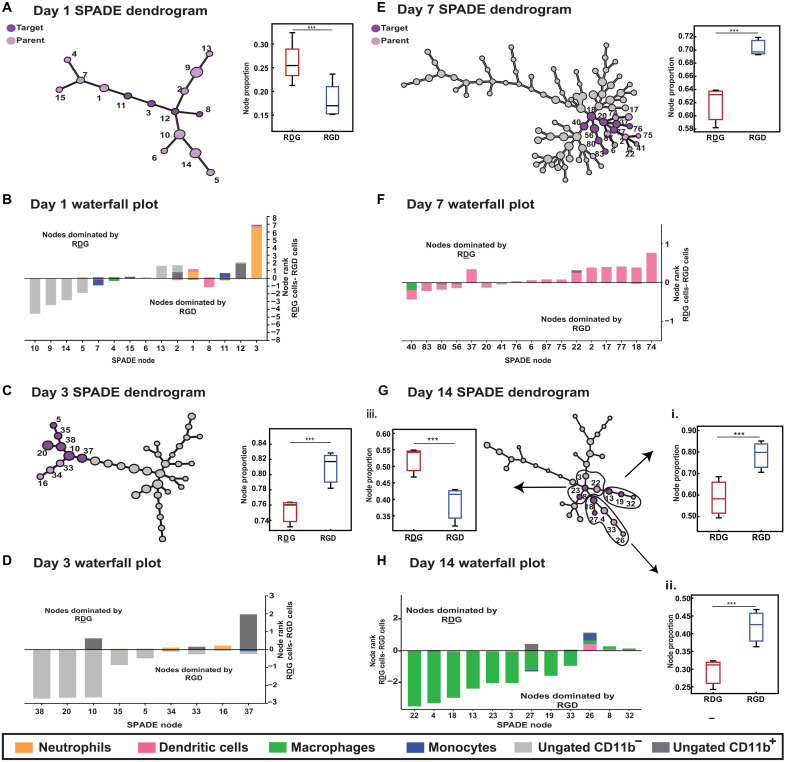
SPADE pseudo-time dendrogram analysis reveals immune cell heterogeneity at early time points and preferential recruitment of DCs to RDG-presenting hydrogels and macrophages to RGD-presenting hydrogels at later time points. SPADE dendrograms are generated from single-cell events infiltrating RDG- and RGD-presenting hydrogels at 1, 3, 7, and 14 (**A**, **C**, **E**, and **G**) dpi. Larger clusters of nodes are identified in which annotated target nodes (dark purple) within a greater parent cluster of nodes (light purple) are significantly different in their recruitment to RGD versus RDG hydrogels at 1, 3, 7, and 14 dpi. Waterfall plots are generated with the SPADE nodes identified in the annotated node clusters for each time point (**B**, **D**, **F**, and **H**) to visualize the immune cell subsets clustered into each of these nodes and whether they are preferentially recruited to RGD- or RDG-functionalized hydrogels. Numbered SPADE nodes on waterfall plots correspond to numbered nodes on SPADE dendrogram. *n =* 3 to 4 hydrogels per experimental group. Statistical analysis includes *t* test multiple comparisons, ***P < 0.001.

To further characterize the cellular and phenotypic plasticity in response to the adhesive ligand, we used X-shift and SPADE as a method to identify nontraditionally gated or rare cell subsets with different biological properties and to infer differences in cellular transition states. After determining the optimal node number via X-shift (density estimation algorithm), we generated SPADE dendrograms composed of single cells extracted from RGD- and RDG-presenting hydrogels (VEGF-loaded and -unloaded) at 1, 3, 7, and 14 dpi (Fig. 5, A to H, and figs. S5 to S8). Dendrograms can be divided into myeloid (CD11b^+^) and nonmyeloid (CD11b^−^) nodes where dark gray nodes depict high expression of CD11b, and light gray nodes indicate those that contain nonmyeloid cells (fig. S5). Frequency of “target” cells (dark purple nodes) with respect to “parent” cells (light purple nodes) were statistically different for RDG and RGD groups and were determined by SPADE. The nodes in light gray did not show differences. The insets represent the proportion of target nodes with respect to parent nodes.

On 1 dpi, fewer distinct subsets of myeloid cells are clustered as opposed to later time points as determined by X-shift (Fig. 5A), suggesting an increase in cell heterogeneity over time (total number of nodes at day 1, 15; day 3, 39; day 7, 93; and day 14, 36). These manual gates were overlaid onto the SPADE dendrograms to display the heterogeneity obscured within manually gated populations in FlowJo (conventional biplot gating; figs. S1 to S3). A single manually gated population may distribute over several nodes in the dendrogram, indicating a heterogeneous population. Cell frequencies of manually gated populations per node were exported, and waterfall plots were generated by subtracting the average value of RGD-functionalized gels from RDG-presenting gels to visualize which nodes are composed of cells primarily from RGD- versus RDG-presenting hydrogels (Fig. 5, B, D, F, and H).

Using our day 1 SPADE dendrogram, we found that a target cluster of nodes (dark purple; nodes 3, 8, 11, and 12) is composed of significantly more cells extracted from RDG-presenting hydrogels than from RGD-presenting hydrogels in relation to the entire dendrogram (light purple) (Fig. 5A, inset, *P* < 0.001). Using the day 1 waterfall plot, we observe that three of those target nodes (nodes 3, 11, and 12) are dominated by RDG-functionalized hydrogels, and those nodes consist primarily of neutrophils and monocytes, respectively (Fig. 5B). The waterfall plot visualization shows that although node 8 is grouped within the target cluster that contains significantly more cells from RDG-presenting hydrogels than those displaying RGD, node 8, in particular, contains primarily DCs, which are preferentially recruited to RGD-presenting hydrogels at day 1 (Fig. 5B).

The day 3 SPADE dendrogram reveals a greater extent of heterogeneity within the immune response to implanted PEG hydrogels, as evidenced by the increased number of nodes (Fig. 5C). SPADE analysis identifies unique target nodes (dark purple) that contain significantly more immune cells, primarily ungated, nonmyeloid (CD11b^−^) cells, from hydrogels presenting RGD adhesive peptide than its scrambled peptide control (Fig. 5C, inset, *P* < 0.001). When loading gels with VEGF, the heterogeneity increases further. SPADE dendrogram at 3 dpi identified a single SPADE node composed of neutrophils (node 15) that contain significantly more cells from RDG-presenting hydrogels (VEGF-loaded) than RGD-presenting hydrogels (VEGF-loaded) when compared to a cluster of nodes that share similar expression profiles for other markers (cluster 3) (fig. S6, inset). Overall, SPADE identified a large number of ungated (unidentified) CD11b^−^ nonmyeloid population in RGD-presenting hydrogels at 1 and 3 dpi. In contrast, cells from RDG-presenting hydrogels presented higher phenotypic heterogeneity, including monocytes and a large number of CD11b^+^ Ly6G^+^ neutrophils.

Day 7 dendrograms exhibited the most heterogeneous cell populations as denoted with a SPADE dendrogram made up of 93 distinct nodes as optimized by X-shift (Fig. 5E), likely due to the various cellular transitions from proinflammatory to proresolution occurring within the inflammatory cascade around this time point. Within this complex immune response, we observed an increase in cells from RGD-functionalized hydrogels in the target nodes (dark purple) (Fig. 5E, inset, *P* < 0.001). Both nodes (dark purple and purple) are dominated by DCs spread among several distinct nodes. Whereas traditional biplot gating strategies lack the functionality to separate out DC subpopulations with a limited panel of surface markers, SPADE analysis enables such discrete distinctions. Several nodes within the parent node contain more cells extracted from RDG-presenting hydrogels (Fig. 5F), highlighting the heterogeneity in the DC response to the adhesive ligand. These results show a marked difference in the myeloid phenotypic transition and frequency accumulation as the response to the adhesive ligand correlated with the observed differences in tissue responses at 7 dpi (Fig. 1B).

At day 14 dpi, overall heterogeneity in the immune response decreased drastically from 93 to only 36 nodes (Fig. 5G). We identified three distinct clusters of nodes (annotated I to III) with varying responses to the presence of adhesive ligand. We found a significant increase in cells recruited to RGD-presenting hydrogels in clusters I and II (Fig. 5G, insets i and ii, *P* < 0.001). The target nodes (dark purple) within the parent clusters (I and II) were composed mainly of cells expressing different macrophage markers. In contrast, cluster III presented more cells expressing monocyte and dendritic markers recruited by RDG-presenting gels (Fig. 5G, inset iii, *P* < 0.001). This is consistent with SPADE dendrograms generated from VEGF-loaded RDG-presenting hydrogels where DCs are present among some annotated nodes at 7 dpi (fig. S7); however, a persistent heterogeneous DC accumulation was observed for both VEGF-loaded RDG- and RGD-presenting hydrogels at 14 dpi (clusters 1 and 2; fig. S8). Together, most annotated nodes at day 14 contained different macrophage subpopulations that were located among multiple nodes dominated by cells preferentially recruited to RGD-functionalized hydrogels. These analyses indicated not only a significant increase in cellular transitions stimulated by the RGD ligand but also an increased adhesion and infiltration of more specific macrophage subsets, consistent with the tissue responses observed (Figs. 3 and 5H).

### Immune cell subset identified by SPADE is sensitive to adhesive cues and alters the functional cytokine environment

**Fig. 6.**
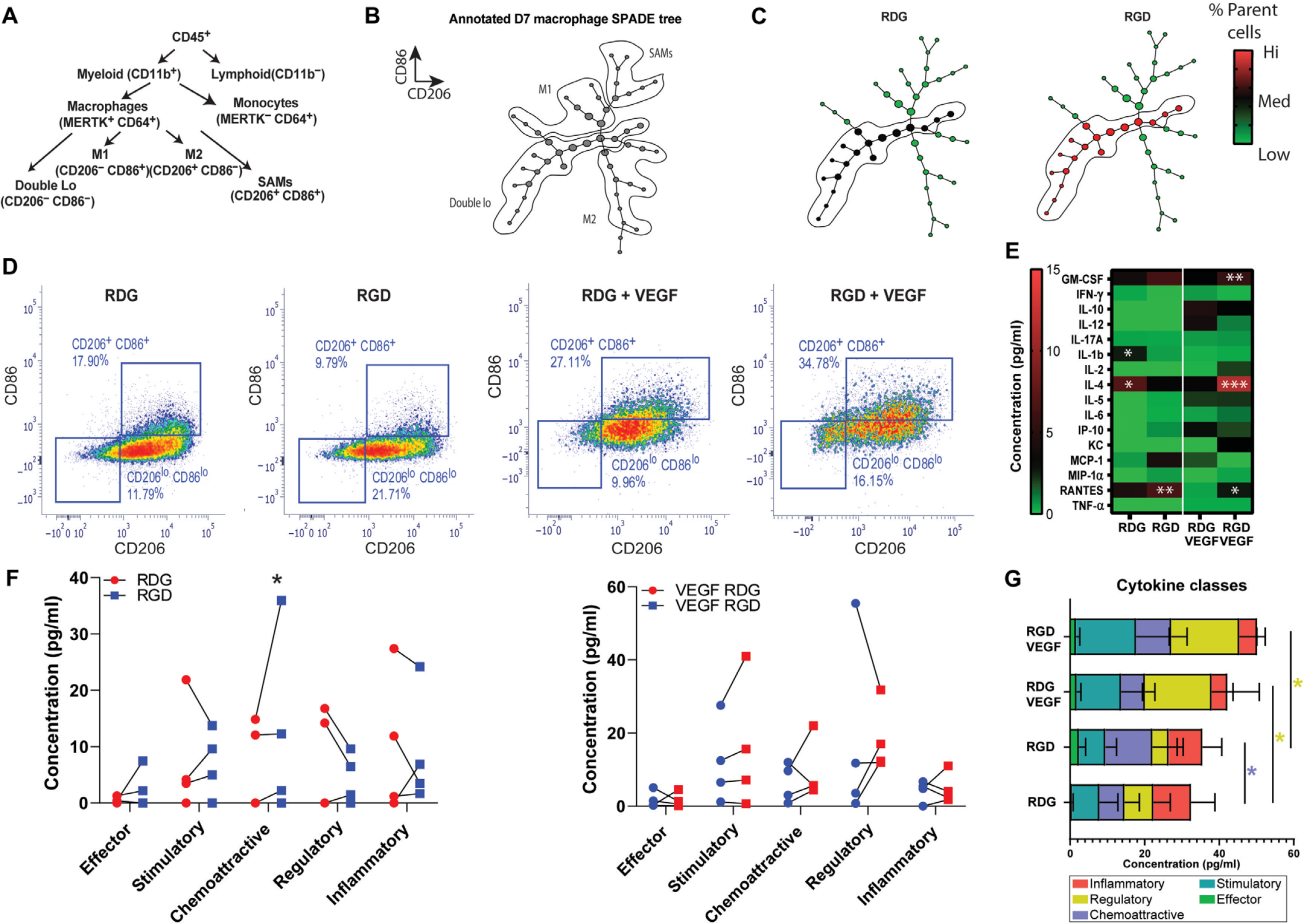
RGD stimulates cytokine secretion in the SPADE-identified macrophage population. (**A**) Classical hematopoietic hierarchy showing the stepwise cell differentiation process. (**B**) Annotated SPADE tree derived from the cytometry flow data at day 7. (**C**) SPADE trees colored for the mean percentage of total number of cells. (**D**) MerTK^+^CD64^+^ CD86^−^ CD206^−^ macrophages were FACS-sorted from RGD- or RDG-functionalized hydrogels with or without VEGF at 7 dpi. Biplots show the cell-sorting strategy used. (**E**) Sorted macrophages were loaded into CodePlex (Isoplexis) chips for multiplexed cytokine analysis. The heatmap shows the median cytokine concentration of sorted double-lo macrophages from RGD- or RDG-functionalized hydrogels with or without VEGF at 7 dpi. (**F**) Additional dot-plot graphs for paired data of cytokine concentration. (**G**) Cytokines grouped into classes for each treatment group. *P < 0.05; ***P < 0.01.

To further evaluate functional heterogeneities underlying macrophage phenotypic transitions in response to RGD adhesive peptide and VEGF, we generated a SPADE dendrogram comprising all CD64^+^ MerTK^+^ macrophages from RGD- and RDG-presenting hydrogels. On the basis of differential CD86 and CD206 expression, we annotated the nodes of the SPADE tree into four distinct subpopulations: M^+/+^ (CD86^hi^ CD206^hi^), M1 macrophages (CD86^hi^ CD206^lo^), M2 macrophages (CD86^lo^ CD206^hi^), and double-lo (CD86^lo^ CD206^lo^) macrophages. The ability of SPADE to identify known populations such as inflammation-promoting (M1) or anti-inflammatory (M2) macrophages was assessed by manually annotating the dendrogram (Fig. 6A). We identified each macrophage phenotype and provided the annotations for the different SPADE regions (Fig. 6B). The SPADE dendrogram suggested that the annotated macrophage subsets (M1 and M2) might transition from an “intermediate” macrophage subset that we refer to as double-lo (Fig. 6B). Low-expressing macrophages for CD86 and CD206 are often overlooked in traditional gating schemes, but SPADE enabled the robust observation and measurement of the accumulation of double-lo macrophages to hydrogels (Fig. 6C). In addition, SPADE determined that the CD86^lo^ CD206^lo^ macrophage subpopulation accumulated preferentially on RGD- compared to RDG-presenting gels for both unloaded and VEGF-loaded gels (Fig. 6C).

To validate the SPADE-based analyses, we used FACS and quantified the accumulation of the sorted double-lo macrophage subpopulation from explanted RGD- or RDG-presenting gels (with or without VEGF). We sorted the macrophage double-lo population from explanted hydrogels and evaluated the differences in cytokine expression signatures (Fig. 6, D and E). The flow cytometry results showed that more double-lo sorted cells accumulated preferentially on RGD-presenting gels compared to RDG-functionalized gels (Fig. 6D). Last, we used protein production profiling to resolve cellular functional heterogeneities as a response to adhesive ligand or VEGF. We detected significant changes in the total production of cytokines for sorted double-lo macrophages from explanted RGD-functionalized gels compared to RDG-presenting gels (Fig. 6E). However, chemoattractive (RANTES, *P* < 0.01) and effector (MIP-1α, ns) cytokines were increased for RGD-presenting gels, whereas regulatory (IL-4, *P* < 0.05) and inflammatory (IL-1β, *P* < 0.05) were decreased, suggesting a critical distinct cell function of the immune cell subset when stimulated by the adhesive ligand (Fig. 6F). Overall, VEGF loading increased the total production of regulatory cytokines (IL-4, *P* < 0.05) and decreased chemoattractive (RANTES, *P* < 0.05) and inflammatory (MCP-1α, *P* <0.05) cytokines compared to unloaded RGD- or RDG-presenting gels. Notably, the synergistic effect of VEGF and adhesive ligand increased stimulatory (GM-CSF, *P* < 0.05), regulatory (IL-4, *P* < 0.001) and chemoattractive (RANTES, *P* < 0.05) cytokine production compared to VEGF-loaded RDG-presenting gels. These results demonstrate the ability of SPADE to identify a subpopulation with a high affinity toward adhesive gels, which functionally alters the in vivo cytokine environment. In addition, the results showed that VEGF further stimulated the total cytokine production (Fig. 6G). Overall, the functional immune landscaping accurately showed that the ligand presentation in the hydrogels resulted in a higher stimulation of overall cytokine production of the SPADE-identified double-lo macrophage subpopulation.

## DISCUSSION

Synthetic hydrogels have great potential to enable a wide range of novel immunotherapies, including treatments for certain cancers, autoimmune diseases, or neurological disorders and technologies to stimulate tissue repair processes (*44*–*48*). Functionalization of hydrogels using a bottom-up approach to leverage immunomodulatory moieties, such as integrin-binding RGD peptides, growth factors (VEGF), or matrix metalloproteinase (MMP)-cleavable peptides, is used extensively to modulate cell adhesion, migration, and proliferation and is particularly important for in vivo vascularization of hydrogel implants and successful wound healing (*37*, *49*). As one of the first responders to injury, macrophages interact with the instructive characteristics of engineered hydrogels and can transition from a large spectrum of proinflammatory phenotypes toward a functionally prohealing phenotype to dictate the outcome of the implant (*11*, *50*). However, the impact of RGD integrin binding on immune cell functions remains unclear—in vivo and in vitro studies have shown contrasting results in the function and polarization of macrophages in response to RGD ligand presentation (*51*, *52*). McWhorter *et al.* (*53*) demonstrated that macrophage transition toward M2-like phenotype is directed by integrin-mediated adhesion and associated with reduced secretion of inflammatory cytokines. Hind *et al.* (*54*) showed that in adhesive hydrogels, M2 macrophages migrate faster and generate higher traction forces than intermediate phenotypes, including M1 macrophages. In contrast, Zaveri *et al.* (*51*) and others reported that RGD integrin–binding promoted macrophage polarization toward M1-like phenotypes and formation of thicker fibrous capsules in vivo. To unlock the full therapeutic potential of synthetic hydrogels, it is essential to understand how immune cells interact with these synthetic materials. Here, we investigated how adhesive peptides presented on engineered hydrogels modulate migration, cytokine secretion patterns, and function of immune cells at the material-tissue interface.

As effector cells, macrophages exhibit substantial phenotypic and functional heterogeneity. Macrophages can exist in a broad spectrum of phenotypes because of their involvement in multiple diverse activities such as homeostasis, inflammation, angiogenesis, and wound healing. However, most of our understanding of macrophage polarization comes from traditional assays (RNA sequencing, bulk enzyme-linked immunosorbent assay, and flow cytometry) and can only estimate overall levels of cytokines or a limited number and fail to characterize the true multi-effector response from immune cells. Upon activation, innate immune cells secrete multiple and diverse effector proteins to carry out protective activities, and the multiplexed detection of all these relevant proteins (>30 cytokines) is necessary to truly characterize the functional phenotype. We engineered hydrogels with equivalent mechanical, biochemical, and structural properties but with different cell adhesion properties by incorporating the adhesive RGD peptide or the nonadhesive scrambled peptide, RDG. Using single-cell cytokine secretome profiling, we showed marked polyfunctional diversity in macrophages isolated from adhesive or nonadhesive gels that showed similar surface marker expression profiles based on multiparametric flow cytometry analyses. Previous work using single-cell proteomics technique on phenotypically similar macrophages (using traditional immunophenotyping techniques) has shown an intrinsic functional heterogeneity, as distinct subpopulations with varying levels of cytokine production were present in unstimulated macrophages (*55*, *56*). Following lipopolysaccharide stimulation, the cosecretion of multiple cytokines per cell (polyfunctionality) was significantly increased, showing a higher fraction of polyfunctional cells.

In our studies, highly polyfunctional macrophages isolated from RDG-presenting gels produced multiple cytokines with heterogeneous functions (inflammatory, effector, chemoattractant, and stimulatory). In marked contrast, macrophages interacting with RGD-functionalized hydrogels presented different functional phenotypes characterized by fewer cytokines per cell with more defined functions (stimulatory and/or chemoattractant). The relationship between macrophage polyfunctionality and macrophage adhesion behaviors has not been previously reported, but we note that differences in PSIs of immune cells have been recently associated with COVID-19 disease severity and treatment outcomes of chimeric antigen receptor (CAR) T-cell immunotherapy (*4*, *47*). Moreover, using intravital imaging and tissue cytokine analyses, we demonstrated that RGD presentation from the hydrogel resulted in different tissue responses such as vascularization, modulated the migratory activity of myeloid cells, and regulated cytokine signature at the tissue-hydrogel interface. Increased secretion of proinflammatory (IL-6 and IL-1β) and anti-inflammatory (VEGF and MIP-1α) cytokines was detected for RGD-functionalized hydrogels compared to RDG-presenting gels, suggesting that integrin binding to biomaterials shifts macrophage polarization toward M1 and M2 phenotypes (*19*). These studies help establish the foundations for designing biomaterials to control the selective recruitment of immune cell subsets and polyfunctional immune functions that determine material integration into host tissue, vascularization, and foreign body reactions.

Single-cell analysis technologies continue to grow and become more widely available with increasingly high number of measured cell features. As a result, there is a strong interest in understanding how the newly observed phenotypic heterogeneity of immune cells regulates inflammatory phase transitions on a more granular level. The lack of important temporal immune cell recruitment and polarization differences between RGD- and RDG-presenting hydrogels was unexpected, given a large body of literature showing that RGD-presenting biomaterials support cell adhesion (*57*). However, these analyses relied on a set of graphs (biplots) or high-dimensional data reduction methods such as UMAP that cannot infer the order of state to state (hierarchy) of cellular transitions or identify rare relevant subpopulations. Unbiased clustering algorithms without compounding user bias and error, such as SPADE, can reveal both low-frequency and transitional immune cells states that would typically be excluded from traditional biplot gating analysis, providing critical insight into how biophysical signals influence immune cell function and enabling intuitive visualization of cellular transition states. Using SPADE and X-shift, we demonstrated significant differential recruitment of myeloid cell subsets to RGD- compared to RDG-presenting hydrogels, which did not fall into traditional biplot gates and were not easily visualized using traditional dimensionality reduction methods. In addition, we found that most macrophage subpopulations distributed among multiple nodes were dominated by cells preferentially recruited to RGD-presenting hydrogels within the dendrogram. Using SPADE analysis, we also showed that macrophage populations with similar traditional phenotypes defined by sets of surface markers in flow cytometry exhibited significant polyfunctional diversity when assessed by single-cell proteomic profiling.

SPADE-based observations suggested an innate heterogeneity and demonstrated that RGD peptides promoted the recruitment of an M1-M2 intermediate macrophage phenotype, CD86^lo^ CD206^lo^ macrophages (double-lo). We performed multiplex proteomics for functionally phenotyping the double-lo macrophage population and observed an increase in the average cytokine production (chemoattractive, stimulatory, effector, and regulatory) as a function of the adhesive ligand. Single-cell proteomics revealed previously unreported adhesion-dependent functional heterogeneity in immune populations defined as relatively homogeneous by traditional surface markers. We expect that this advanced, in-depth temporal study of biomaterial immune response will inform the future development of biomaterial-based strategies for immunomodulation applications. Furthermore, combining single-cell proteomics with hierarchical cell clustering algorithms (SPADE) has demonstrated the potential to comprehensively understand the heterogeneous polyfunctionality of macrophages in response to biophysical cues. These innovative combinatorial immunophenotyping methodologies provide a new platform for analyzing the functional activity of traditionally overlooked immune subpopulations.

## MATERIALS AND METHODS

### PEG hydrogel fabrication

Four-arm PEG macromer end-functionalized with maleimide (PEG-4MAL >95% functionalization, Laysan Bio) at 4.5% w/v was used for all hydrogel formulations. PEG macromers were functionalized with RGD peptide (GRGDSPC) or RDG scrambled peptide control (GRDGSPC) and cross-linked with the cysteine-flanked peptide VPM (GCRDVPMSMRGGDRCG) (AAPPTec) in 0.5 M MES buffer (pH 5.5). The final concentration of RGD or RDG was 1.0 mM, and the final concentration of VEGF was 10 μg/ml. The cross-linker concentration was based on the concentration of nonreacted maleimide groups remaining on PEG macromers. To generate preformed hydrogels for intravital imaging, the hydrogel was cast in a 4-mm-diameter circular silicon mold. After cross-linking, hydrogels were incubated at 37°C for 15 min and then swelled in phosphate-buffered saline (PBS) for at least 30 min. For studies where hydrogels were fluorescently tagged, RDG or RGD was dissolved in sodium bicarbonate and tagged with Alexa Fluor 405 *N*-hydroxysuccinimide ester (Life Technologies) according to the manufacturer’s recommendation. Tagged RDG or RGD was combined with unlabeled peptide in a 1:3 ratio for conjugation to PEG-MAL.

### Subcutaneous implant model

All animal procedures were conducted according to protocols approved by the Georgia Institute of Technology Animal Care and Use Committee. Male C57BL/6J mice (8 to 12 weeks) were used for subcutaneous implant studies. Mice were anesthetized with vaporized isoflurane at 5% concentration and maintained under anesthesia at 1 to 3%. The animal’s dorsal skin was shaved, depilated, and sterilized with chlorhexidine and 70% isopropanol. All animals received a single dose of sustained release buprenorphine (1.2 mg/kg injected intraperitoneally) before implantation. A 23G needle was first inserted into the subcutaneous space where hydrogels were to be implanted. Hydrogel precursors were mixed in a tube and rapidly pulled into a 31G insulin syringe for subcutaneous injection. One hydrogel (25-μl volume) was placed on each side of the spine (two total hydrogels per animal). Animals were euthanized by CO_2_ inhalation for flow cytometry analysis at 1, 3, 7, or 14 dpi. Subsequent immunophenotyping data analysis was performed on the same set of macsamples, including biplot gating, UMAP, X-shift, and SPADE.

### DSWC model

Male C57BL/6J or B6.129P-Cx3cr1tm1Litt/J (CX3CR1^GFP/+^) mice (8 to 12 weeks) were used for DSWC studies. A sterile DSWC (APJ Trading Co.) was placed on the mouse dorsum as previously described (*58*). Briefly, mice were anesthetized with vaporized isoflurane at 5% concentration and maintained under anesthesia at 1 to 3%. The dorsal skin was shaved, depilated, and sterilized with 70% ethanol and chlorhexidine. One of the window chamber titanium frames was fitted on the underside of a double-layered skinfold on the back of the mouse. A 12-mm-diameter circular area of epidermis and dermis was removed from the top of the skinfold using surgical microscissors, revealing the underlying vasculature. Sterile saline was superfused on the exposed tissue throughout surgery to prevent desiccation. The upper titanium frame was placed on the top side of the skinfold, secured to the underlying frame, and sutured to the surrounding tissue. The exposed subreticular dermis was flushed with sterile saline and sealed with a sterile glass window layer (a circular coverslip). For flow cytometry studies, two hydrogels (one RDG and one RGD, 15-μl volume each) were placed on top of the exposed subcutaneous tissue immediately after surgery. For intravital imaging studies, 1 day after surgery (day 1), the coverslip was removed, and two hydrogels were placed on top of the exposed tissue. The window chamber was resealed with a new sterile coverslip. Animals were euthanized by CO_2_ inhalation for flow cytometry analysis at 3 dpi.

### Immunophenotyping by flow cytometry

Hydrogels were explanted from the subcutaneous space, weighed, and placed in cold PBS containing calcium and magnesium. Hydrogels were washed for 30 min on a shaker to remove nonadherent cells. Adherent cells were isolated by digesting hydrogels with collagenase type 1A (1 mg/ml) at 37°C for 30 min and further disaggregated with a cell strainer to ensure a single-cell suspension. Single-cell suspensions were stained for flow cytometry analysis in 3% fetal bovine serum/PBS according to standard procedures and analyzed on a FACSAria IIIu flow cytometer (BD Biosciences). The following antibodies were used for immunophenotyping: BV421- or BV510-conjugated anti-CD11b (BioLegend), anaphase-promoting complex (APC)– or BV510-conjugated anti-Ly6C (BioLegend), PerCP-Cy5.5–conjugated anti-CD11c (BioLegend), APC-Cy7–conjugated anti-Ly6G (BioLegend), BV711-conjugated anti-CD64 (BioLegend), phycoerythrin (PE)–conjugated anti-MerTK (BioLegend), PE-Cy7– or BV605-conjugated anti-CD206 (BioLegend), and fluorescein isothiocyanate (FITC)–conjugated anti-CD86 (BioLegend). Staining using BV dyes was performed in the presence of Brilliant Stain Buffer (BD Biosciences). Positivity was determined by gating on fluorescence minus one control. Absolute quantification of cell numbers was performed by adding 25 μl of AccuCheck counting beads to flow cytometry samples (Thermo Fisher Scientific).

### High-dimensional analysis of flow cytometry data

#### Uniform Manifold Approximation and Projection

UMAP is a nonlinear dimensionality reduction algorithm able to embed high-dimensional data into a space of two or three dimensions. Cells are visualized in a scatterplot, where points that are closer together can be considered more similar. Before UMAP dimensional reduction, each flow cytometry sample was manually pregated to select single cells. The gated FCS (flow cytometry standard) files were imported into Python 3.7 using fcsparser Pandas 2.5 according to (*59*). Each channel, except for FSC and SSC, was normalized by applying arcsinh transformation with a cofactor of 150 to transform fluorescence data into a fold-channel scale. A UMAP representation was generated on MATLAB for each time point (days 1, 3, 7, and 14) with cells extracted from RGD- and RDG-presenting hydrogels.

#### X-shift

The 26 April 2018 version of VorteX was downloaded, and manually gated single-cell events from the day 1, day 3, day 7, and day 14 flow cytometry panels were uploaded into the VorteX clustering environment with four RDG and four RGD samples on day 1, while days 3, 7, and 14 all had three RDG and three RGD samples. The imported settings were as follows: minimal Euclidean length of the profile: 1.0, import maximum: none, and merge all files from each flow cytometry panel into one dataset. FSC, SSC, CD11b, MerTK, CD64, CD86, CD206, Ly6C, CD11c, and Ly6G were selected for clustering. After the data were imported, the clustering settings used are as follows: numerical transformation: arcsinh (x/f), *f* = 5.0, noise threshold: 1.0, feature rescaling: none, normalization: none, distance measure: Euclidean distance, clustering algorithm: X-shift (gradient assignment), density estimate: N nearest neighbors (fast), number of neighbors for density estimate (*K*): from 150 to 10, with 15 steps, and number of neighbors for mode finding (*N*): determine automatically. After clustering was completed, the cluster number was calculated using the elbow point method and was determined to be *K* = 100, *K* = 60, *K* = 50, and *K* = 50 for the day 1, day 3, day 7, and day 14 panels, respectively. This corresponded to an optimal number of clusters of 15, 39, 93, and 36 for days 1, 3, 7, and 14, respectively. The same method was applied to find the optimal number of clusters for flow cytometry data from VEGF-loaded RGD- and RDG-functionalized hydrogels at days 3, 7, and 14 after implantation. The optimal number of clusters was found to be 245, 311, and 114 for these time points, respectively (*K* = 50 for all time points).

#### Spanning-tree progression analysis of density-normalized events

SPADE analysis was performed on single-cell events from the day 1, day 3, day 7, and day 14 flow cytometry panels, respectively, with four RDG and four RGD samples on day 1, while days 3, 7, and 14 all had three RDG and three RGD samples in MATLAB. The raw median fluorescence intensity values were transformed to a hyperbolic arcsine (arcsinh) scale with a cofactor of 150. The target number of nodes was adjusted to 15, 39, 93, and 36 for the day 1, day 3, day 7, and day 14 panels, respectively, as informed by X-shift. FSC, SSC, CD11b, MerTK, CD64, CD86, CD206, Ly6C, CD11c, and Ly6G per respective flow cytometry panel were included in the clustering result. The parameters were as follows: Max allowable cell in pooled down sampled data: 50,000, outlier density: first percentile of local densities (LDa) of all cells, target density: fixed number of cells remain 20,000. Neighborhood size = median min dist.*: 5, local density approximation factor: 1.5, algorithm: K-means. After the SPADE dendrogram is generated, manually gated populations made in FlowJo were overlaid using a MATLAB script to indicate cell frequencies of the manually gated populations in each node of the generated SPADE Tree. Fluorescence intensity and cellular frequencies of each node from each individual were exported for further analysis. The same method was applied to generation of SPADE dendrograms from single cells extracted from VEGF-loaded hydrogels (RGD- and RDG- functionalized) at days 3, 7, and 14 after implantation. Dendrograms were generated with optimal node numbers of 245, 311, and 113 (for days 3, 7, and 14) as determined by X-shift.

#### Heatmap of fluorescence intensity values

Fluorescence intensity values from the resulting SPADE trees for each flow cytometry panel were used to generate z scores to produce a heatmap per fluorophore per node.

#### Node frequency differences between RDG and RGD hydrogels

Average cell frequency values per cell type node were arranged in descending order to establish ordered difference (RDG-RGD). Average cell differences above 0 indicate decrease in cell frequency proportions in RGD hydrogels, whereas differences below 0 indicate an increase in cell frequency in RGD hydrogels compared to control microgels. The individual frequencies of the top five and bottom five nodes after being ranked in descending order were further analyzed and compared between RDG and RGD with appropriate statistical test.

### Intravital confocal microscopy

Mice were anesthetized with vaporized isoflurane at 5% concentration and maintained under anesthesia at 1 to 3%. The glass coverslip was removed, and sterile saline was administered to the exposed dorsal tissue to prevent desiccation. The anesthetized mouse was secured to the microscope stage in a custom adapter placed on top of a heating block to maintain body temperature. Intravital confocal microscopy was performed using a 20× water immersion objective (numerical aperture = 1.0) fixed to an inverter on a Zeiss LSM710 NLO microscope. Time-lapse z-stack images were acquired at each hydrogel edge. A step size of 5 μm was used in the *z* direction. Videos of 25 to 30 min were acquired at each location, with a time step of 30 s, and a total of two to three videos were acquired for each hydrogel, and one video was acquired in distal tissue without a hydrogel.

### Intravital imaging analysis

For 3D analysis in Imaris (Bitplane), time-lapse images were acquired adjacent to the implant to visualize immune cell distribution in the close surrounding tissue. Cells expressing CX3CR1-GFP were identified in Imaris using the surface tool. CX3CR1^+^ surfaces were identified by smoothing with a 2.77-μm grain size and a threshold value of 7.71 on absolute intensity. Touching objects were split using a seed point diameter of 10.4 μm with a quality threshold above 3.53. CX3CR1^hi^ versus CX3CR1^lo^ cells were discriminated by assigning half of all cells to each group based on maximum fluorescence intensity in the CX3CR1-GFP channel. The hydrogel surface was identified using the surface tool with a 10-μm grain size and a manually selected threshold value on absolute intensity. To calculate the distance between cells and the hydrogel surface, a distance transformation was applied to the hydrogel surface, and the minimum distance of each cell was recorded. To track cell activity over time, cells were identified in Imaris using the spots tool. Estimated diameter was set to 8.00 μm with background subtraction enabled and an automatic threshold on quality. Tracks were selected for analysis if they lasted at least 300 s. Tracks were set to a maximum distance of 10.0 μm and a max gap size of 3 μm. Because statistical comparisons were made on a single-cell basis, an equal number of cells was used for analysis of each video. The minimum number of detected track was 73. Therefore, for videos with more than 73 tracks, we randomly selected 73 tracks for analysis.

### Cytokine analysis in vitro

Hydrogels and tissue were collected and snap-frozen using liquid nitrogen. In preparation for the inmunoassay, proteins were lysed using nondenaturing lysis buffer supplemented with Halt protease inhibitor (Thermo Fisher Scientific) and centrifuged at 10,000*g* for 15 min at 4°C to remove debris. Supernatant was collected and stored at −80°C until analysis. Lysates were analyzed using the Millipore Mouse Cytokine 32-plex Assay (Millipore) on a Magpix multiplexing machine (Luminex) according to the manufacturer’s instructions.

### Isoplexis secretome analysis

#### Single-cell IsoCode analysis

Hydrogels were explanted from the subcutaneous space at day 7 and digested as described for immunophenotyping by flow cytometry. Single-cell suspensions were stained with Zombie Red viability dye, PE-conjugated anti-MerTK, and FITC-conjugated anti-CD64. CD64^+^MerTK^+^ macrophages were sorted on a FACSAria IIIu flow cytometer (BD Biosciences) and collected into FACS tubes with complete RPMI 1640 media. Sorted macrophages were pooled together per treatment group (*n* = 3) and stained with membrane stain (provided in kit) for 10 min at 37°C for cell identification. After incubation, stained cells were centrifuged at 300*g* for 10 min and resuspended in complete RPMI 1640 media at a final cell density of 1000 cells/μl. A total of 30,000 macrophages were loaded into the IsoCode chips per treatment group, and chips were loaded into IsoLight instrument to measure all targeted single-cell cytokine secretions (IsoCode Mouse Adaptive Immune panel). IsoSpeak software was used for automated quantitative measurements.

#### Bulk CodePlex analysis

Hydrogels were explanted from the subcutaneous space at day 7. Single-cell suspensions were stained with the following antibodies: Zombie Red Viability dye, PE-conjugated anti-MerTK, FITC-conjugated anti-CD64, PE-Cy7-conjugated anti-CD206, and APC-conjugated anti-CD86. CD64^+^MerTK^+^ CD206^lo^ CD86^lo^ cells were sorted on a FACSAria IIIu flow cytometer (BD Biosciences). Sorted cells were lysed using nondenaturing lysis buffer and centrifuged at 14,000*g* for 10 min. Supernatant was collected and stored at −80°C until analysis. All lysate samples had a final concentration of 0.09 mg/ml total protein (Pierce 660 nm Protein Assay Kit, Thermo Fisher Scientific) before loading into CodePlex chips. Lysis buffer was used for background measurements. Codeplex chips were inserted into the IsoLight instrument to measure the secreted cytokine profile of each sample for all targeted cytokines (CodePlex Mouse Adaptive Immune panel). IsoSpeak software was used for automated quantitative measurements.

### Statistical analysis

Data are presented as means ± SD unless otherwise noted. All statistical analysis was performed in GraphPad Prism software. For comparisons of two groups, paired *t* test was used. For comparison between changes in gel weight across time, one-way ANOVA with Tukey posttest was used for multiple comparisons. For grouped analyses, two-way ANOVA with Sidak’s posttest was used for multiple comparisons. *P* < 0.05 was considered statistically significant.

## Notes

View/request a protocol for this paper from *Bio-protocol*.
