# Supplementary material for: Analyzing immune response to engineered hydrogels by hierarchical clustering of inflammatory cell subsets
Source: Sci Adv. 2022 Feb 25;8(8):eabd8056. doi: 10.1126/sciadv.abd8056 (PMC8880784; doi:10.1126/sciadv.abd8056)
Supplement: Supplementary file 1 — Figs. S1 to S10 [file sciadv.abd8056_sm.pdf]

Science Advances
AAAS

Supplementary Materials for

Analyzing immune response to engineered hydrogels by hierarchical clustering of inflammatory cell subsets

Marc A. Fernandez-Yague, Lauren A. Hymel, Claire E. Olingy, Claire McClain, Molly E. Ogle, José R. García, Dustin Minshew, Sofiya Vyshnya, Hong Seo Lim, Peng Qiu, Andrés J. García, Edward A. Botchwey*

*Corresponding author. Email: edward.botchwey@bme.gatech.edu

Published 25 February 2022, *Sci. Adv.* **8**, eabd8056 (2022)
DOI: 10.1126/sciadv.abd8056

This PDF file includes:

Figs. S1 to S10

Supplementary Material

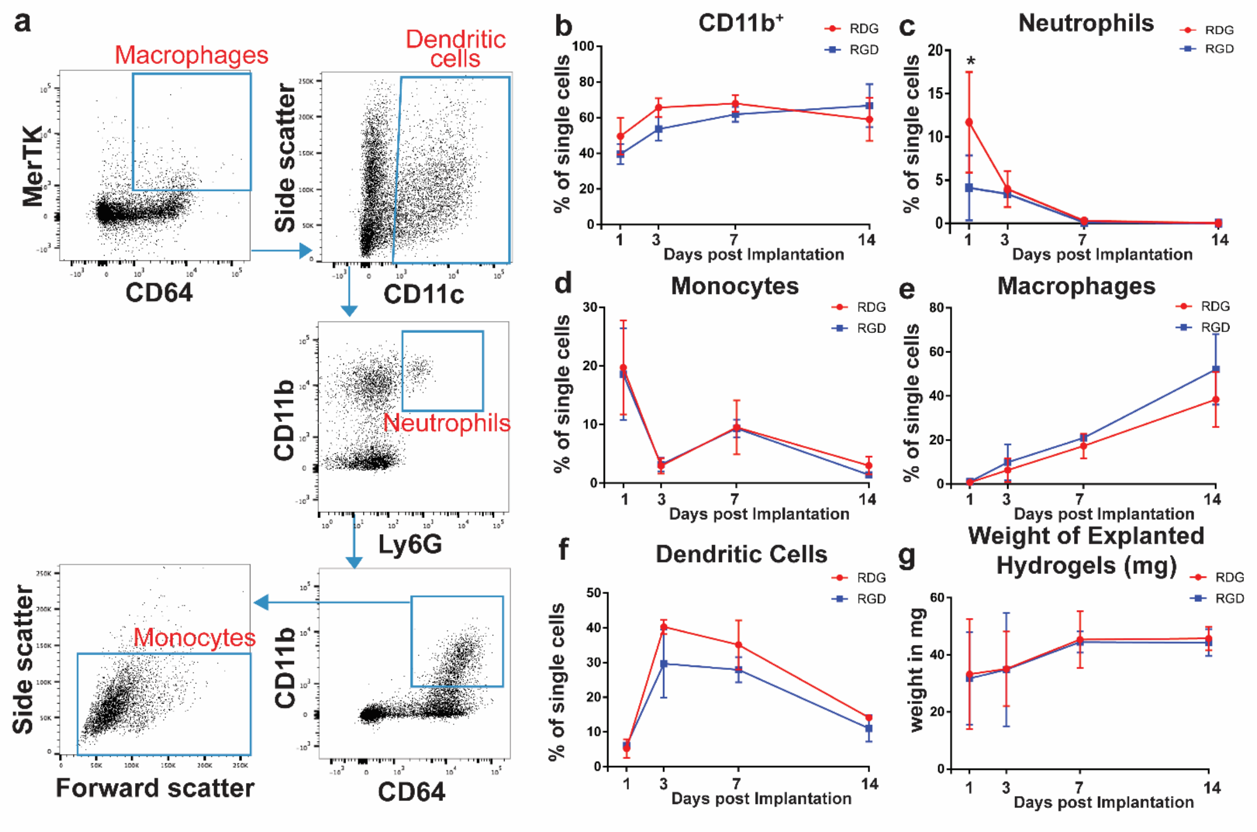

Supplementary Figure 1. Traditional bi-plot gating strategy for flow cytometry lacks multiparameter visualization necessary for heterogeneous immunophenotyping. Flow cytometry gating strategy and immune cell subset quantifications at days 1, 3, 7, and 14 dpi into subcutaneous model. At indicated timepoints, hydrogels were extracted and analyzed by flow cytometry. (a) Traditional, sequential bi-plot gating strategy to identify myeloid immune cell subsets. (b-f) Identified immune cell subsets as a percentage of all single cells extracted from RDG- (red) or RGD- (blue) functionalized hydrogels at each timepoint. (g) Weight of explanted hydrogels (mg) at each of the indicated timepoints. Data presented as mean ± SD. Statistical comparisons performed via paired t-tests between groups at each timepoint. n=3-7 animals per timepoint, internally controlled design.

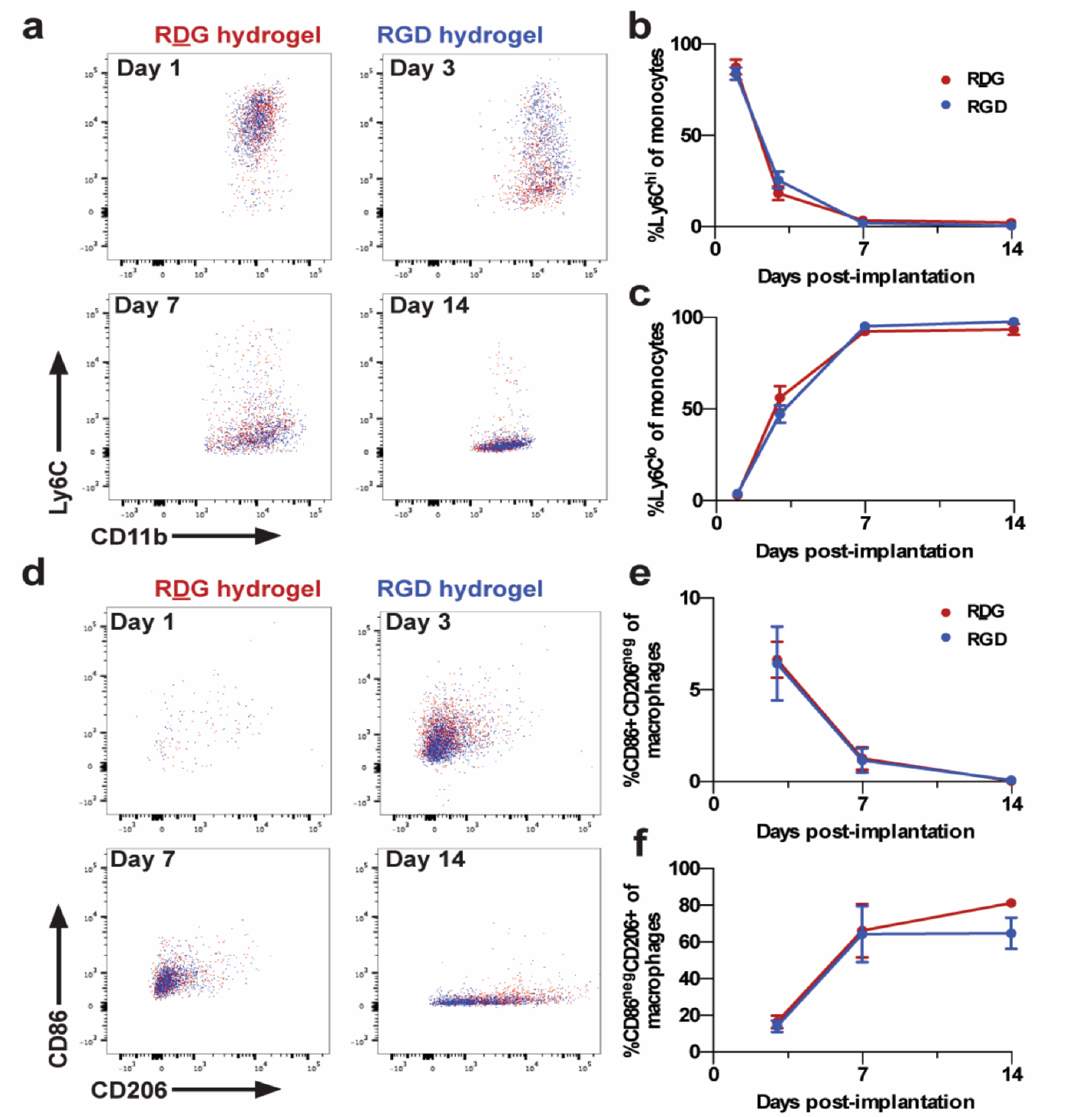

Supplementary Figure 2. Temporal evolution of monocyte and macrophage subsets adhered to PEGhydrogels. (a) Representative flow cytometry dot plots of CD64+SSC^{lo} monocytes collected from explanted RDG (red) or RGD (blue) hydrogels. Quantification of the frequency of (b) classical Ly6C^{hi} or (c) non-classical Ly6C^{lo} monocytes, expressed as a frequency of total monocytes. (d) Representative flow cytometry dot plots of MerTK+CD64+ macrophages collected from explanted RDG (red) or RGD (blue) hydrogels. Quantification of (e) CD86+ CD206^{neg} M1 and (f) CD86^{neg}CD206+ M2 macrophages, expressed as a frequency of total macrophages. Data presented as mean ± SEM n= 3-7 animals per timepoint, internally controlled design.

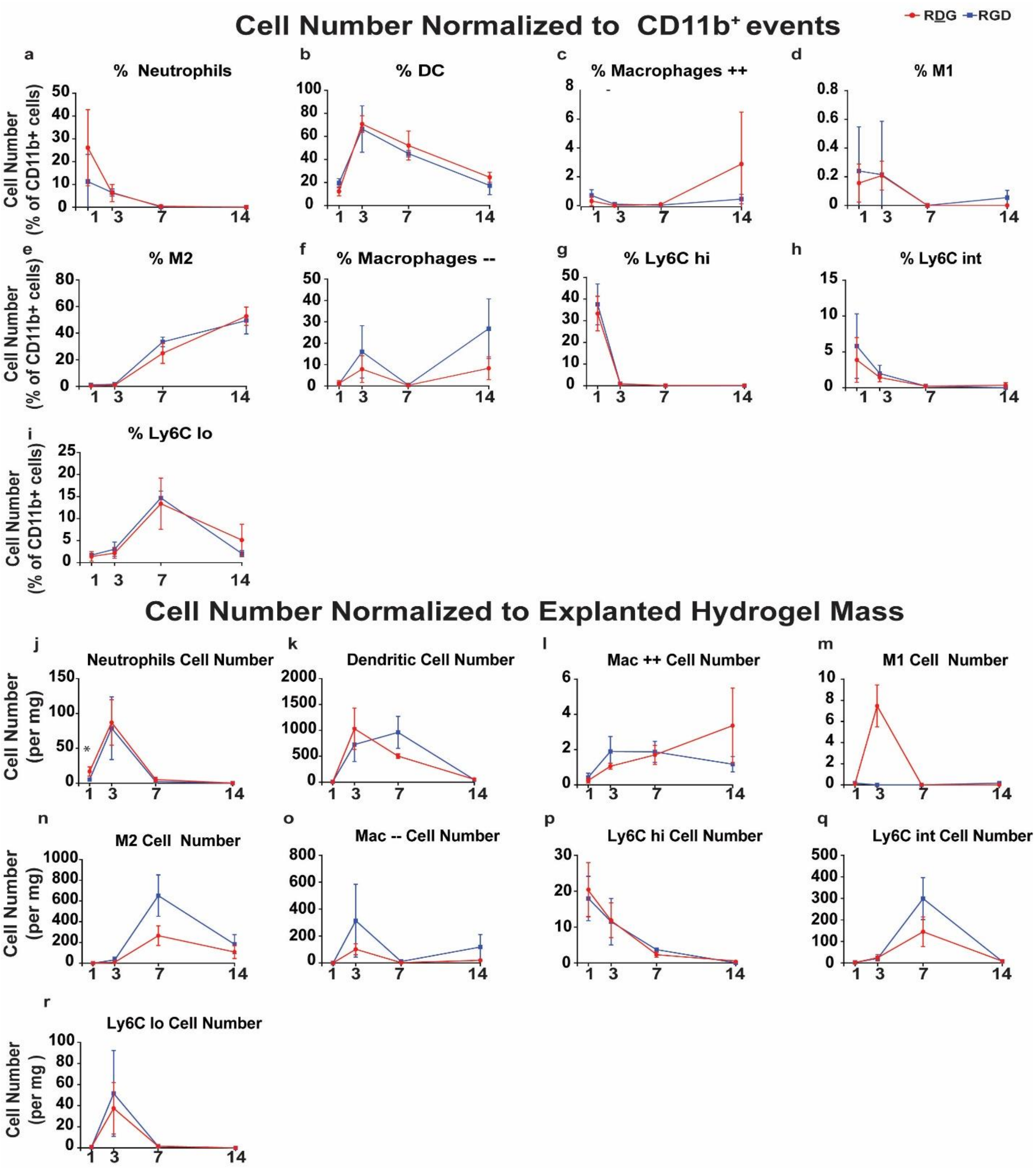

Supplementary Figure 3. Immune cell quantifications from flow cytometry analysis normalized to total myeloid cells and weight of explanted hydrogel. (a-i) Immune cells collected from explanted hydrogels presented as a percentage of CD11b+ myeloid cells at days 1, 3, 7, and 14 dpi into subcutaneous space. Immune cell subsets quantified include (a) neutrophils, (b) dendritic cells, (c) macrophages^{++} (CD86+ CD206+), (d) M1 macrophages (CD86+ CD206^{neg}), (e) M2 macrophages (CD86^{neg}CD206+), (f) macrophages^{--} (CD86- CD206-), (g) Ly6C^{hi} monocytes, (h) Ly6C^{int} monocytes, and (i) Ly6C^{lo} monocytes. (j-r) Immune cell subsets presented as cell number per mg of explanted hydrogel at days 1, 3, 7, and 14 post implantation. Data expressed as mean ± SD. Statistical analysis conducted with paired t-test between groups at each time point, *$p<0.05$, n=3-4 animals per timepoint, internally controlled design.

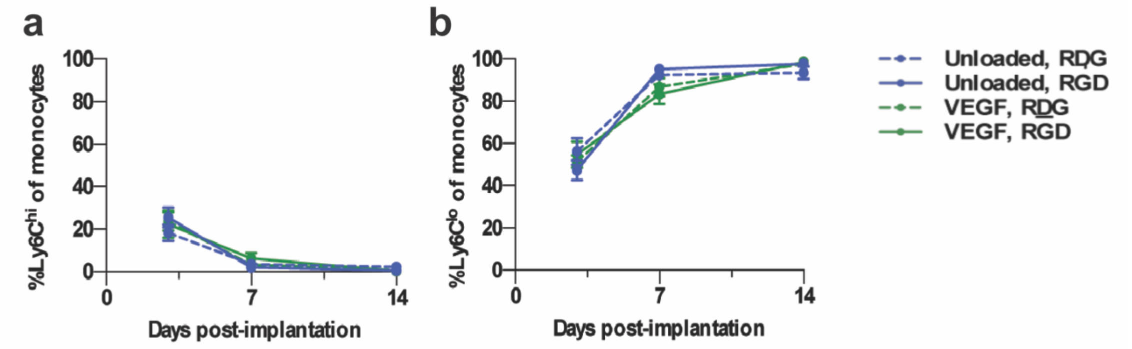

Supplementary Figure 4. Temporal evolution of monocyte subsets around PEG hydrogels delivering VEGF. Quantification of the frequency of (a) classical Ly6C^{hi} and (b) non-classical Ly6C^{lo} monocytes, expressed as a frequency of total monocytes. Data presented as mean ± S.E.M. n=3-11 animals per timepoint with internally controlled design.

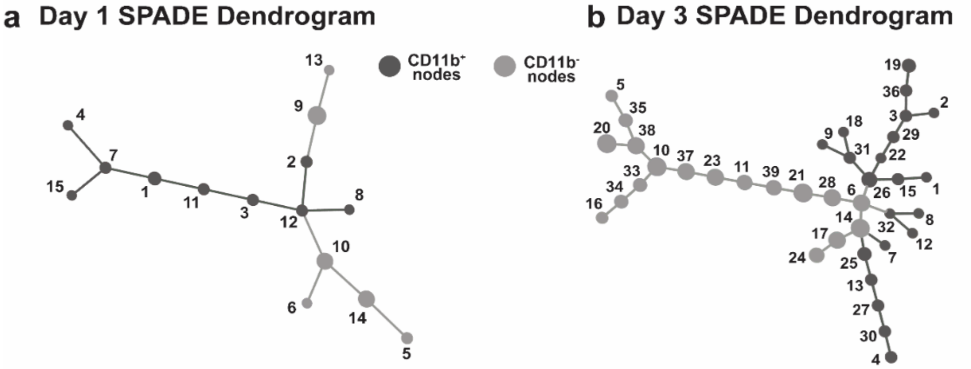

Supplementary Figure 5. Differential expression of CD11b at day 1 and day 3 post hydrogel implantation. SPADE dendrograms are generated from single cell events infiltrating both RDG- and RGD-functionalized hydrogels at day 1 (a) and day 3 (b) post injection into the subcutaneous implant model. To visualize the proportion of myeloid vs non-myeloid cells comprising the dendrogram, SPADE nodes are annotated based on expression of CD11b where dark gray nodes represent CD11b^{+} nodes and light gray nodes represent CD11b^{-} nodes.

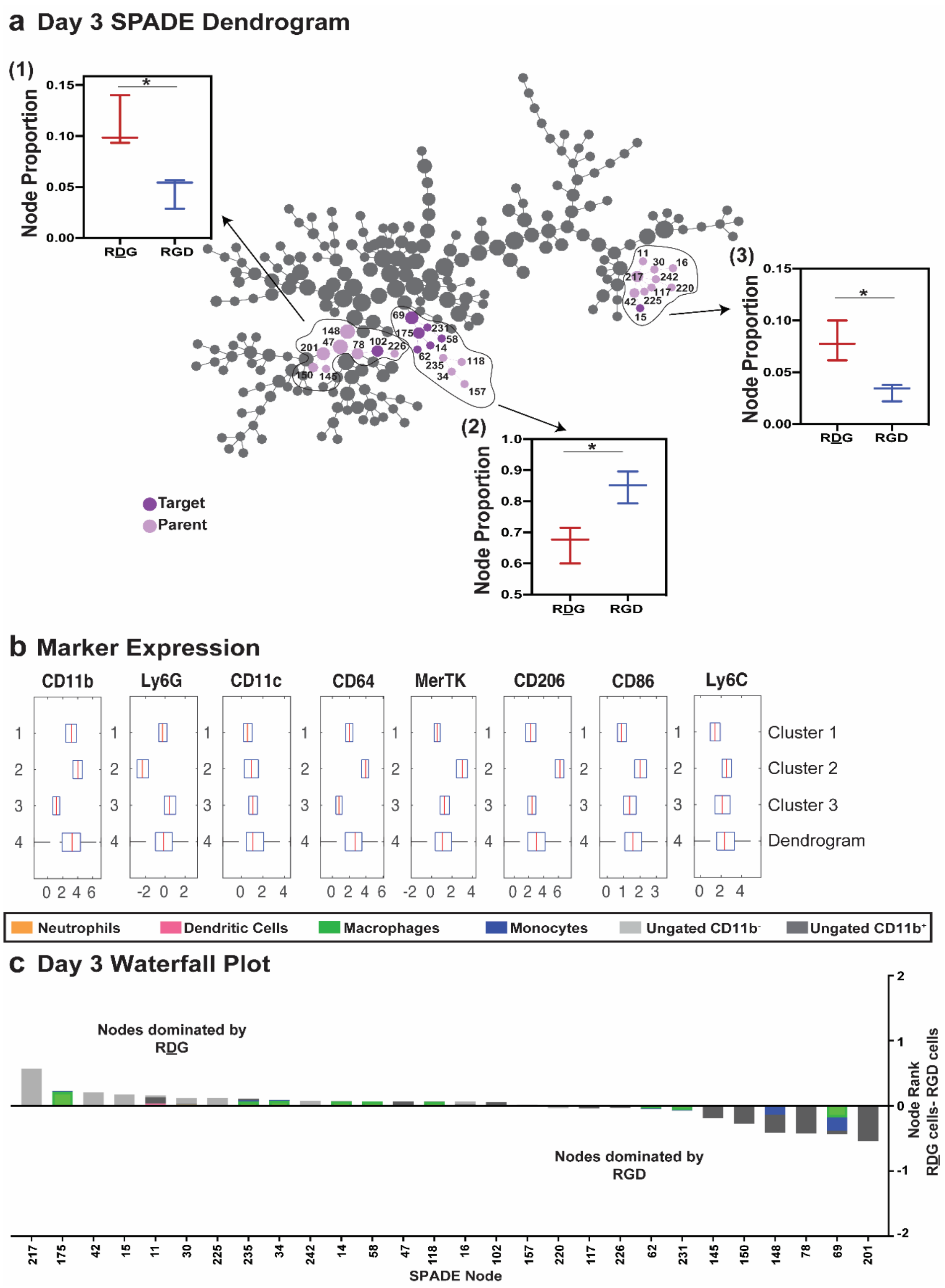

Supplementary Figure 6. SPADE analysis with cells adhered to VEGF-loaded gels at day 3 post implantation identifies macrophage cluster biased to adhesive ligand functionalized hydrogel. (a) SPADE dendrogram is constructed from single cell events infiltrating VEGF-loaded RDG- and RGD-functionalized hydrogels at day 3 post hydrogel implantation into the subcutaneous space. Three distinct clusters of nodes (circled in black) are identified in which annotated 'target' nodes (dark purple) within a greater 'parent' cluster of nodes (light purple) are significantly different in their recruitment to RGD- vs RDG- functionalized VEGF-loaded hydrogels. (b) Marker expression values characterize the phenotype of each cluster of nodes annotated in the SPADE dendrogram as compared to the marker expression plotted for the entire dendrogram. (c) A waterfall plot is generated with the SPADE nodes identified in each of the annotated node clusters to visualize the immune cell subsets clustered into each of these nodes and whether they are preferentially recruited to RGD or RDG-functionalized hydrogels loaded with VEGF. Numbered

SPADE nodes on waterfall plot corresponds to numbered nodes on SPADE dendrogram. Data expressed as minimum, median, and maximum. Statistical analysis includes t-test multiple comparisons, *p<0.05. n=3 animals, internally controlled design.

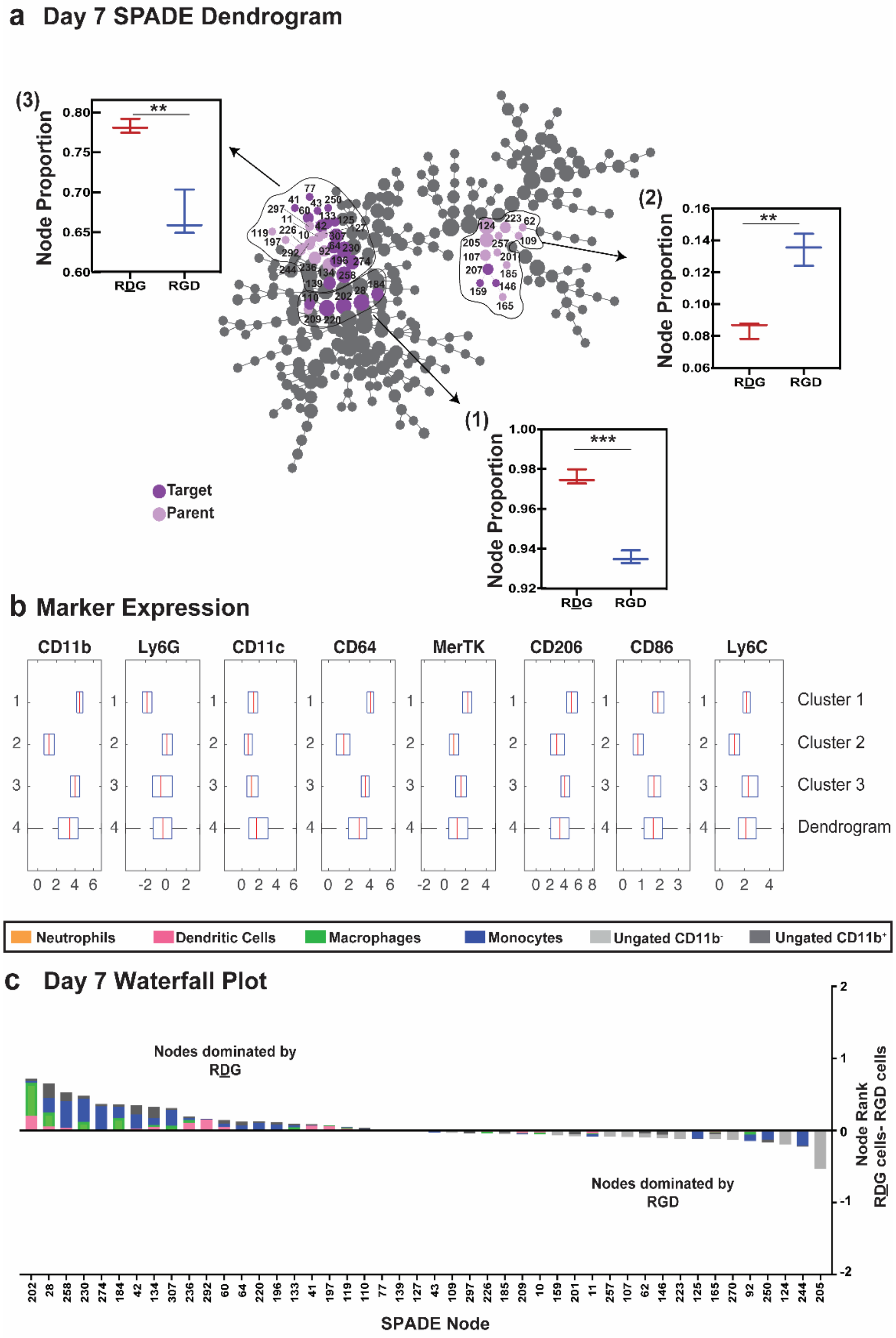

Supplementary Figure 7. VEGF-loaded hydrogels induce increased monocyte and macrophage recruitment to RDG hydrogels at day 7 post implantation. (a) SPADE dendrogram is constructed from single cell events infiltrating VEGF-loaded RDG- and RGD- functionalized hydrogels at day 7 post hydrogel implantation into the subcutaneous space. Three distinct clusters of nodes (circled in black) are identified in which annotated 'target' nodes (dark purple) within a greater 'parent' cluster of nodes (light purple) are significantly different in their recruitment to RGD- vs RDG- functionalized VEGF-loaded

hydrogels. (b) Marker expression values characterize the phenotype of each cluster of nodes annotated in the SPADE dendrogram as compared to the marker expression plotted for the entire dendrogram. (c) A waterfall plot is generated with the SPADE nodes identified in each of the annotated node clusters to visualize the immune cell subsets clustered into each of these nodes and whether they are preferentially recruited to RGD or RDG-functionalized hydrogels loaded with VEGF. Numbered SPADE nodes on waterfall plot corresponds to numbered nodes on SPADE dendrogram. Data expressed as minimum, median, and maximum. Statistical analysis includes t-test multiple comparisons, **p<0.01, ***p<0.001. n=3 animals with internally controlled design.

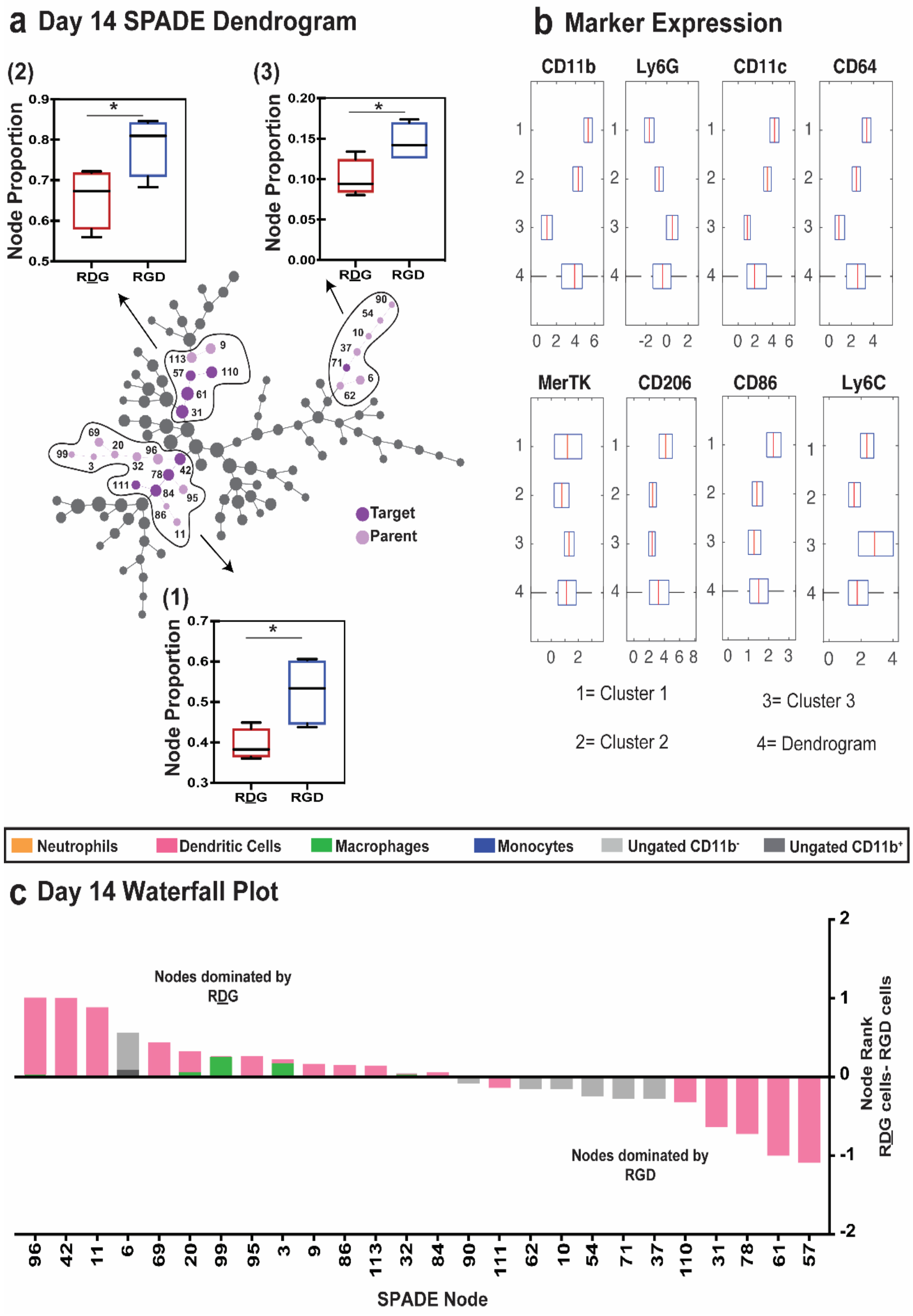

Supplementary Figure 8. Heterogeneous dendritic cell recruitment to both RDG and RGD hydrogels at day 14 in response to VEGF delivery. (a) SPADE dendrogram is constructed from single cell events

infiltrating VEGF-loaded RDG- and RGD- functionalized hydrogels at day 14 post hydrogel implantation into the subcutaneous space. Three distinct clusters of nodes (circled in black) are identified in which annotated 'target' nodes (dark purple) within a greater 'parent' cluster of nodes (light purple) are significantly different in their recruitment to RGD- vs RDG- functionalized VEGF-loaded hydrogels. (b) Marker expression values characterize the phenotype of each cluster of nodes annotated in the SPADE dendrogram as compared to the marker expression plotted for the entire dendrogram. (c) A waterfall plot is generated with the SPADE nodes identified in each of the annotated node clusters to visualize the immune cell subsets clustered into each of these nodes and whether they are preferentially recruited to RGD or RDG-functionalized hydrogels loaded with VEGF. Numbered SPADE nodes on waterfall plot corresponds to numbered nodes on SPADE dendrogram. Data expressed as minimum, 25th percentile, median, 75th percentile, and maximum. Statistical analysis includes t-test multiple comparisons, *p<0.05. n=4 animals with internally controlled design.

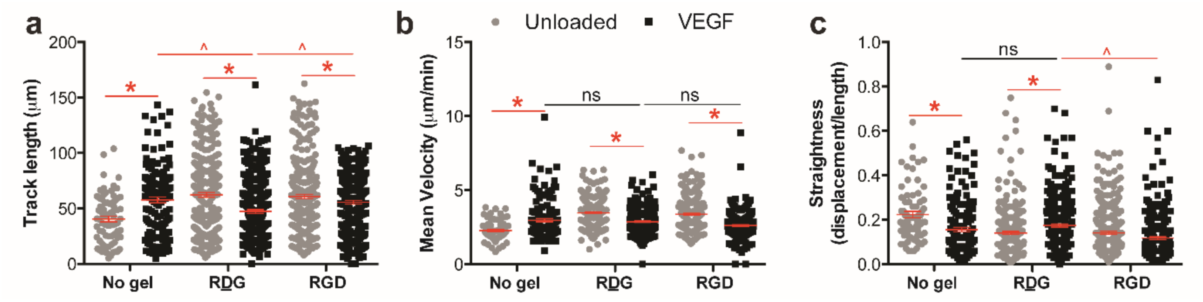

Supplementary Figure 9. Quantification of myeloid cell migration around PEG hydrogels delivering VEGF. (a) Track length (in μm), (b) mean velocity (in μm/minute), and (c) track straightness (the displacement normalized to the path length) of CX3CR1+ cells moving in tissue without a hydrogel or around hydrogels functionalized with RDG or RGD. Red bar indicates the mean. *p<0.05 compared to unloaded hydrogel or tissue, ^p<0.05 compared to RDG hydrogel by Kruskal-Wallis. n>146 cells across 4 mice (2 mice received unloaded hydrogels, 2 mice received VEGF hydrogels).

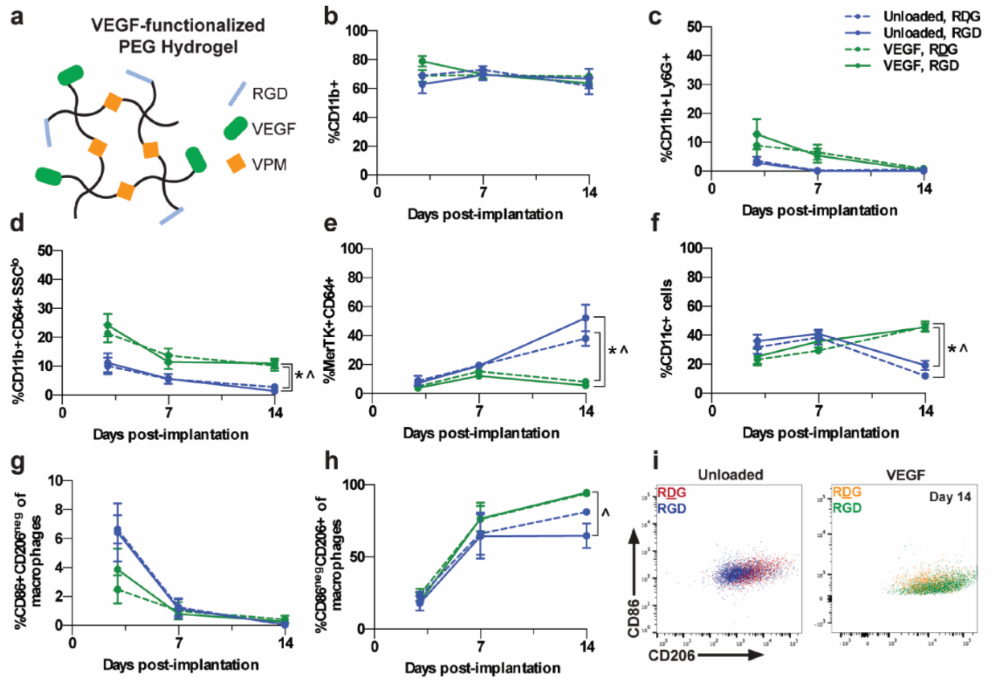

Supplementary Figure 10. Effect of controlled VEGF delivery on myeloid cell response to hydrogel implantation. (a) Schematic representation of 4 arm PEG-MAL macromers functionalized with RGD (or control RDG) peptides and crosslinked with a protease-degradable peptide, VPM. (b-h) Immune cell subsets quantified by flow cytometry at days 3, 7, and 14 post hydrogel injection into subcutaneous space. All quantifications are normalized to percentage of single cells between unloaded hydrogels (blue) and VEGF-loaded hydrogels (green), functionalized with either RGD (solid line) or RDG (dotted line) peptides. Immune cells analyzed include CD11b+ myeloid cells (b), neutrophils (c), monocytes (d), macrophages (e), dendritic cells (f), M1 macrophages (g), and M2 macrophages (h). Representative flow cytometry dot plots from which M1 and M2 macrophages were identified at day 14 post hydrogel implantation (i). Data expressed as mean ± SEM *$p<0.05$ compared to unloaded RDG hydrogel, ^$p<0.05$ compared to unloaded RGD hydrogel by one-way ANOVA at the indicated time point. n=3-11 hydrogels per group.
